# Environmental characteristics associated with the presence of the Spinetail devil ray (*Mobula mobular*) in the eastern tropical Pacific

**DOI:** 10.1371/journal.pone.0220854

**Published:** 2019-08-07

**Authors:** Nerea Lezama-Ochoa, Martin A. Hall, Maria Grazia Pennino, Joshua D. Stewart, Jon López, Hilario Murua

**Affiliations:** 1 Inter-American Tropical Tuna Commission, Bycatch Program, La Jolla, San Diego, CA, United States of America; 2 AZTI-Tecnalia, Marine Research Division, Pasaia, Spain; 3 Instituto Español de Oceanografía (IEO), Vigo, Spain; 4 Scripps Institution of Oceanography, La Jolla, San Diego, CA, United States of America; 5 The Manta Trust, Dorchester, Dorset, United Kingdom; 6 International Seafood Sustainability Foundation (ISSF), Washington, DC, United States of America; Tanzania Fisheries Research Institute, UNITED REPUBLIC OF TANZANIA

## Abstract

In the eastern Pacific Ocean, the tropical tuna purse-seine fishery incidentally captures high numbers of five mobulid bycatch species; all of which are classified as mortalities by the Inter-American Tropical Tuna Commission due to uncertainties in post-release mortality rates. To date, the factors (operational or environmental) leading to the capture of these species by the fishery have not been well studied. Here, we developed Generalized Additive Models for fisheries observer data to analyze the relationships between the presence/absence of *Mobula mobular* bycatch and oceanographic conditions, the spatial and temporal variability in fishing location, and the set type (associated with dolphins, free-swimming tuna schools or floating objects). Our results suggest that chlorophyll concentration and sea surface height are the most important variables to describe the presence of *M*. *mobular* in conjunction with geographic location (latitude and longitude) and set type. Presence of the species was predicted in waters with chlorophyll concentrations between 0.5–1 mg·m^-3^ and with sea surface height values close to 0; which indicates direct relationships with productive upwelling systems. Seasonally, *M*. *mobular* was observed more frequently during December-January and August-September. We also found the highest probability of presence observed in School sets, followed by Dolphin sets. Three areas were observed as important hotspots: the area close to the coastal upwelling of northern Peru, the area west to Islands Colon Archipelago (Galapagos) and the area close to the Costa Rica Dome. This information is crucial to identify the mobulids habitat and hotspots that could be managed and protected under dynamic spatial management measures to reduce the mortality of mobulid rays in the eastern Pacific purse-seine fishery and, hence, ensure the sustainability of the populations of these iconic species.

## Introduction

Understanding the distribution of highly mobile marine species is necessary for developing effective conservation and management strategies, especially for those species that are impacted by fisheries [1, 2].

Mobulid rays are one of the groups most heavily impacted by global fisheries pressure due to the demand for their meat, skin and gill plates [3–5]. This demand has resulted in dramatic increases in fishing effort for mobulid rays in some regions [6–8]. At least 13 fisheries in 12 countries specifically target mobulids. Moreover, mobulids are also caught in many other fisheries worldwide as bycatch [8, 9]. For example, mobulids are caught as target species in small-scale fisheries using driftnets, gillnets, harpoons, gaffs, traps, trawls, and longlines. As bycatch, mobulids are caught in large-scale fisheries using driftnets, longlines, trawls and purse seines [8, 10–12].

The tropical tuna purse-seine fishery is one of the fisheries that capture the highest number of mobulid species in the eastern Pacific Ocean [9]; compared with other oceans and fishing gear types [8, 11]. In 2012, the total incidental bycatch of mobulid species reached 3,297 individuals [13]. As there is still limited data on the post-release survival of these species, all mobulid bycatch numbers are considered as mortality by the Inter-American Tropical Tuna Commission (IATTC) even if released [9].

Despite the low frequency of mobulid captures per set [8, 13], the large number of sets combined with the distribution of purse seiner fishing effort worldwide make the bycatch rates of purse seiners considerable important to conservation. The bycatch of these species in various fisheries in conjunction with the low reproductive potential contribute to their declining stocks [5, 12, 14, 15].

All mobulid species have recently been added to Appendix II of the Convention on International Trade in Endangered Species (CITES) and to Appendices I and II of the Convention of Migratory Species (CMS) [5, 16] in order to meet regional conservation goals as well as curb international trade in mobulid products.

In the case of the eastern Pacific Ocean, the Inter-American Tropical Tuna Commission (IATTC) have adopted and implemented a conservation and management measure (C-15-04) to reduce the mortality of mobulid species in the Convention Area [17]. This measure prohibits retaining onboard, transshipping, landing, storing, selling, or offering for sale any part or whole carcass of mobulid rays taken by purse seiners.

The Spinetail Devil Ray (J.P. Müller and Henle, 1841) (*Mobula mobular;* recently reviewed and changed from *Mobuja japanica* [18]) is one of the mobulid species caught most frequently in the purse-seine fishery in the eastern Pacific Ocean [8, 9, 13]. *M*. *mobular* is listed as “Near Threatened” globally (after lumping with *M*. *japanica*) and “Endangered” within the Mediterranean Sea by the International Union for Conservation of Nature (IUCN) Red List of Threatened Species (http://www.iucnredlist.org/). The species is mobile, distributed circumglobally in tropical and subtropical waters, and can be found in both coastal and oceanic pelagic waters [19, 20]. Therefore it is possible that the large-scale movement patterns of *M*. *mobular* between productive regions and the seasonal aggregations at specific locations can be explained by food availability (i.e. seasonal distribution patterns of euphausiids). The water column dynamics may also affect their vertical migrator behavior and distribution [6, 16, 19, 21, 22]. Previous studies demonstrate a preference for shallow (less than 50 m depth) and warm (more than 20°C) waters in Mexico, the Mediterranean Sea, and the southwest Pacific Ocean [19]. However, there is still a major knowledge gap in the relationship between the spatial distribution of this species and the environmental conditions at large spatial oceanic scales, such as the Pacific Ocean.

Species Distribution Models (SDMs) are useful tools to study statistical relationships between species occurrence or abundance and environmental covariates and to predict potential habitat preferences [23]. Species Distribution Models have been used in terrestrial and freshwater habitats, but their use has been limited in oceanic waters. The challenge of collecting adequate sample sizes from across large marine areas and the difficulty in correctly identifying species across multiple data sources have lead to a lesser use of these models [24].

Among the different Species Distribution Models methods (e.g. regression models, machine learning), Generalized Additive Models (GAMs) are used most often to model the distribution and environmental preferences of large marine species [25–27]. These models have been used for target and non-target species in commercial fisheries using different gears: longliners, trawlers or purse-seiners [26, 28–32], but never applied to mobulid rays. In the case of the purse-seine fishery, Generalized Additive Models (GAMs) have been applied to model the habitat preferences of diverse bycatch species [13, 25, 26, 33–40]; but again have not yet been applied to mobulid rays datasets.

Studies evaluating the environmental factors affecting the distribution of mobulids are mostly based on visual census or aerial surveys [41–45], sightings [1, 46] or tagging data [47–54], and studies on devil rays (excluding the more frequently studied manta rays) are scarce in general [6, 10]. With respect to *M*. *mobular*, only a few studies [19, 20, 55] describe their environmental preferences because most studies are limited by low sample sizes and constrained spatial extent. In that sense, SDMs could help to improve the knowledge about the spatial distribution of *M*. *mobular* using a large dataset and the most important environment variables which characterize the ecology of the species.

The Inter-American Tropical Tuna Commission (IATTC) observer database offers a unique opportunity to model the occurrence of *M*. *mobular* using fishery-dependent data at large spatial-temporal scales. The purse seine observer programs provide more than 80% coverage of purse seine vessels (class 6) operating throughout the EPO from 1993 until 2017. This program offers unprecedented data richness to answer questions about distribution and habitat use of *M*. *mobular*. Improving knowledge about the environmental preferences and spatial distribution of *M*. *mobular* will contribute to the implementation of effective fisheries management measures in the eastern Pacific Ocean [16, 23].

This study explored the environmental variables that influence the spatial distribution of *Mobular mobular* bycatch in the eastern Pacific Ocean tuna purse-seine fishery. The study used a Generalized Additive Model and oceanographic variables derived from satellite data to address the seasonal and spatial variation of this species. We hypothesize that the presence of *M*. *mobular* is directly related to the oceanographic conditions of the eastern Pacific Ocean and, specifically, with the most important upwelling systems.

## Material and methods

### Study area

The purse-seine fishery for tunas in the eastern Pacific Ocean is primarily concentrated in the tropical water of the IATTC management area, which extends from 50°S to 50°N. The characteristic of this area is influenced by the Peru and California ocean currents which flow from the north and south of the area, respectively. The presence of the eastern Pacific warm pool, located along the coast of southwestern Mexico and Guatemala and the equatorial cold tongue at about 120°W also influence the characteristic of the study area (S1 Fig) [56, 57].

The surface chlorophyll concentration, an index of phytoplankton biomass in near-surface waters, is high in the winter around i) the Gulfs of Tehuantepec, Papagayo and Panama driven by three Central American wind jets and ii) in the Peru coastal upwelling system (S1 Fig)[56, 57]. Conversely, during summer, chlorophyll is highest along the equator due to upwelling driven by the southeast trade winds and at the Costa Rica Dome where the thermocline is shallow (S1 Fig) [56].

### *Mobula mobular* data

The data used in this study is from the Inter-American Tropical Tuna Commission (IATTC) Convention Area (limited by parallel 50°N, 50°S, the meridian 150°W and American Pacific coastline), also known as the eastern tropical Pacific Ocean (ETP).

*Mobula mobular* bycatch data were collected by the Agreement on the International Dolphin Conservation Program (AIDCP) onboard observer program that employ both, observers from the National Observer Program and IATTC observers, from 2005 to 2015, monitoring captures from large purse seine vessels (> 363 t carrying capacity-Class 6) using three types of fishing modes or set: Dolphin sets, Floating object sets and School sets.

Dolphin sets are sets on tunas associated with dolphin, School sets are sets on unassociated tuna schools and Floating object sets are sets on tunas associated with encountered natural objects (Log) or/and objects deployed by the fishers, called drifting Fish Aggregating Devices (dFADs) [9]. Since the late 1990s, most floating-object sets are estimated to have been made on FADs (IATTC 2018). Dolphin and School sets are normally made during daylight hours, while FAD sets are mostly made at dawn [13].

Bycatch data has been collected by the AIDCP on purse seine vessels (Class 6) that operate in the IATTC Convention Area [13]. The AIDCP observer program collects data on: i) the vessel’s route and activity, ii) environmental parameters (wind speed, current speed and sea surface temperature), iii) fishing operations, estimated catch of target species, tuna discards and bycatches (in biomass or number); iv) size distribution of tuna catch, discards and bycatch and v) Floating objects characteristics and/or activities (natural and Fish Aggregating Devices (FADs)) during a tuna set.

The identification of the non-target species is one of the most challenging issues for the program; as observers, sometimes, do not have direct access to the individuals or sufficient time. To improve the identification of the bycatch to the species level, in 2005 the IATTC expanded its species code list to include many more species so that species-specific bycatch could be recorded for many families and genera.

### Oceanographic data

For each fishing set (date and position for the years 2005–2015), the following oceanographic variables were extracted (Table 1): daily sea surface temperature (SST; in °C), daily sea surface height (SSH; in cm), monthly phytoplankton (Phy; in mg·m-^3^), monthly chlorophyll (Chl; mg·m-^3^), daily salinity (Sal; in PSU), daily eddy kinetic energy derived from altimetry (Eke, in m^2^ s-^2^), daily heading and speed of the current derived from UV (Heading; degrees; vel; m/s;), monthly oxygen concentration (O2; mg/l), and monthly Nitrate (Ni; mg/l) (Table 1). All variables were collected at 1/4° spatial resolution using python routines and motu-client from the E.U. Copernicus Marine Environment Monitoring Service (CMEMS) (http://marine.copernicus.eu/), which provides information on the physical state, variability and dynamics of the ocean and marine ecosystems worldwide. Environmental variables were selected and downloaded from the “Ocean product” section from Copernicus database (http://marine.copernicus.eu/services-portfolio/access-to-products/). The variables for each product were selected based on the parameters of interest (year, month, resolution). Each variable information (NC file) was downloaded following the instructions from the website through the use of *phyton* scripts (see an example in S1A Table). Once the *nc* file was downloaded, it was opened using R (see S1B Table). Then, the information for each variable (year, month, day, position) was matched with the information from our database. Finally, all the variables were merged in the same R *dataframe*, which was used to run the models. For the variables with daily resolution, the date (day, month, year) and position (longitude, latitude) of each single set was matched with the date and position of the corresponding oceanographic variables (SST, SSH, Salinity, Eke and Heading) extracted from the satellite data. In the case of variables with monthly resolution, the date (month and year) and position of the set was matched with the position and date of the oceanographic variables (Phy, Chl, O2 and Ni).

**Table 1:** Summary of the environmental variables obtained from Copernicus Marine Environment Monitoring Service (CMEMS): Variable acronym and Name, Unit, Average value, Minimimum value, Maximum value, and Spatial and Temporal resolution.

Variables acronym	Variable name	Units	Average	Min	Max	Spatial resolution	Temporal resolution
**Depth**	Depth	m	3732.411	6476.665	45.357	30 arc-seconds	**-**
**Distance to the coast**	Distance	Km*1000 (Euclidean distance)	8.907	0.059	23.026	5 arcmin	**-**
**SST**	Sea Surface Temperature	°C	25.276	16.690	29.636	0.25°	Daily
**Sal**	Salinity	psu	34.371	26.943	36.453	0.25°	Monthly
**SSH**	Sea Surface Height	m	0.246	-0.001	0.627	0.25°	Daily
**Chl**	Chlorophyll concentration	mg.m-3	0.217	0.024	1.830	0.25°	Monthly
**Phy**	Phytoplankton	mg.m-3	1.611	0.427	15.369	0.25°	Monthly
**O2**	Oxygen Concentration	mg/l	209.613	193.605	252.102	0.25°	Monthly
**Ni**	Nitrate	mg/l	4.785	0.000	20.173	0.25°	Monthly
**Vel**	Velocity	m/s	0.247	0.001	1.161	0.25°	Monthly
**Ke**	Kinetic energy	m/s	0.046	0.000	0.674	0.25°	Monthly
**Heading**	Direction of the current	degrees	213.341	0.000	359.849	0.25°	Monthly

Depth and distance from the coast were obtained as rasters (ASCII format) from Global Marine Environmental Datasets (GMED) (http://gmed.auckland.ac.nz/download.htmlf). Bathymetry values were obtained from General Bathymetric Chart of the Oceans (GEBCO, 08 Digital Atlas) (British Oceanographic Data Centre, UK, www.gebco.net) (depth; m; 30 arc-seconds resolution). Land distance or distance to the nearest land cell (water cells only) was calculated using euclidean distance (distance; km x 100 (euclidean distance); 5 arcmin resolution) (Table 1). Both rasters were imported to R software [58] with the *rasters* package [59] and the depth and distance values matching the position date of species bycatch data were extracted to use in the model. The type of set and spatial-temporal variables such as latitude, longitude, year and month were also considered as potential variables in the model.

### Statistical analysis

Generalized Additive Models (GAMs) [60], were fitted to identify the spatial-temporal dynamics and environmental characteristics associated with the presence of *Mobula mobular* habitat in the eastern Pacific Ocean for 2005–2015. GAMs are semi-parametric extensions of generalized linear models (GLMs) [60]. These models were chosen over generalized linear models as are capable of capturing non-linear relationships by fitting smoothing functions to predictor variables [34, 38].

The presence/absence of *M*. *mobular* was considered as the dependent variable. Data for 1270 presences and 260,002 absences were included in the analyses.

Spatial (latitude and longitude), temporal (month, year), the type of purse-seine set (Dolphin vs. Floating objects vs. School sets) and oceanographic variables were considered as independent variables for the model. The general structure of the GAM is:

$$g(\mu i)=\alpha +f_{1}(X_{1i})+f_{2}(X_{2i})+f_{3}(X_{3i})\ldots .+f_{n}(X_{ni})$$

where *g* is the link function (logit for binomial family), *μi* is the expected response variable (presence-absence), *α* is the intercept, *f*_*n*_ are smooth functions (thin plate regression splines), and *X*_*n*_ are the covariates [60].

The degrees of freedom of the smooth functions were restricted for each explanatory variable to avoid additional over-fitting. Thus, the number of basis functions (k) was associated with each smooth term was set to k = 6 for the main effects and to k = 20 for the interaction effects [61, 62]. Each GAM was fitted using (i) thin plate regression splines for non-linear covariates, except for monthly variation, where a cyclic cubic regression spline was used to account for a cyclical effect [63] and (ii) a two-dimensional thin plate regression spline surface to account for spatial effects (latitude, longitude) of each fishing set [38, 64].

In order to avoid overfitting and reduce correlation and collinearity between variables, two measures were considered. First, all predictor covariates were examined using Pearson’s rank correlation [65]. Pairs of variables with high correlation values (Pearson correlation r > 0.6) were identified and only one of the correlated pair was included in the modelling process [38]. Second, multicollinearity among the predictor variables was assessed by calculating the variance inflation factor (VIF) with a cut-off value of 5 [32, 64] using the function *corvif* of the AED package in R [66]. Because of high correlation, the four pairs of covariates: 1) “phytoplankton-chlorophyll”, 2) “sea surface temperature-oxygen”, 3) “sea surface temperature-sea surface height” and 4) “eddy kinetic energy-velocity of the current” were not considered at the same time in the models. The correlation and collinearity analyses are shown in S2 Fig.

Thirteen candidate final GAM models were considered from on all possible combinations of covariates. Each candidate model was fitted using a binomial family with a logistic link function. A forward step-wise variable selection procedure was applied to establish the models, which consists of building the null model (only the overall mean as a predictor variable) and then adding a new covariate to check its contribution to the model [27]. Covariate contributions were evaluated using model explained deviance and studying their significance (based on p-value). Only significant covariates (p < 0.05) and those with large relative contributions to explained deviance were included in the next step of model. The best fit model of the 13 candidates was selected as the final model according to the lowest Akaike information criterion (AIC) value and the highest explained deviance [67, 68].

A cross-validation was applied with a k-fold partitioning method (with k = 5) to assess model performance [23, 69]. This methodology consists of splitting data into two different sets: one set used to model the relationship between presence-absence data and the environmental variables (80% of data), called the training data, and the other set used to assess the quality of predictions, called the testing data (20% of data) [33, 38]. Model predictions were evaluated by calculating the Area Under the receiver-operating Curve (AUC) and the mean True Skill Statistic (TSS) [70]. The AUC measures the ability of the model to correctly predict where a species is present or absent [71]. The AUC provides a threshold independent measure of overall model accuracy ranging from 0 to 1, where values around 0.5 indicate the prediction is as good as random and values around 1 indicates perfect prediction [72]. AUC is tabulated in a confusion matrix indicating the true positive (TP), false positive (FP), false negative (FN) and true negative (TN) predictions. We obtained from the confusion matrix the Specificity, which indicates the percentage of absences correctly predicted, and Sensitivity, which indicates the percentage of the presences correctly predicted [24].

The TSS is another accuracy index but is threshold dependent and not affected by the size of the validation set [24]. The TSS index ranges from -1 to +1, indicating 0 as no predictive skill and is calculated as sensitivity plus specificity minus 1 from the confusion matrix [24]. Model validation was performed using the *PresenceAbsence* package [73]. Finally, the residuals were tested for spatial autocorrelation using the Moran's I test of the *spdep* package in R [74]. This index ranges between -1 (showing high-dispersion) to 1 (high-correlation). A zero value would indicate a random spatial pattern [68].

Lineplots based on observed values were created to identify possible areas of importance for *M*. *mobular* (based on the longitudinal gradient) in relation with the most important oceanographic variables obtained from the final model.

In this study GAMs were implemented as a modeling algorithm to apply a Species Distribution Model (SDMs). As usual in this context, the main aim is to first estimate the relationship between the studied species and environmental variables, and secondly using the estimated relationship to predict where a species is likely to be present. Areas with higher probability were defined as hotspots that in this case indicate area with higher presence of the studied species. Spatial prediction maps of the average and standard deviation of presence of *M*. *mobular* were created to identify possible hotspots of the species in the study area [38, 68].

Map of the average was done by predicting at all data points in the dataset and then averaging predictions by 1 degree area; excluding set type and month.

Spatial prediction maps by fishing type and period were also created fitting the final model that included set type and period and then averaging prediction over all set types and periods. Three periods were considered: Period 1 (February-May), Period 2 (June-September) and Period 3 (October-January) based on the main oceanographic process of the eastern Pacific. All the models and predictions were done using *predict*.*gam* function from the *mgcv* package [75] in R.

## Results

**Fig 1:**
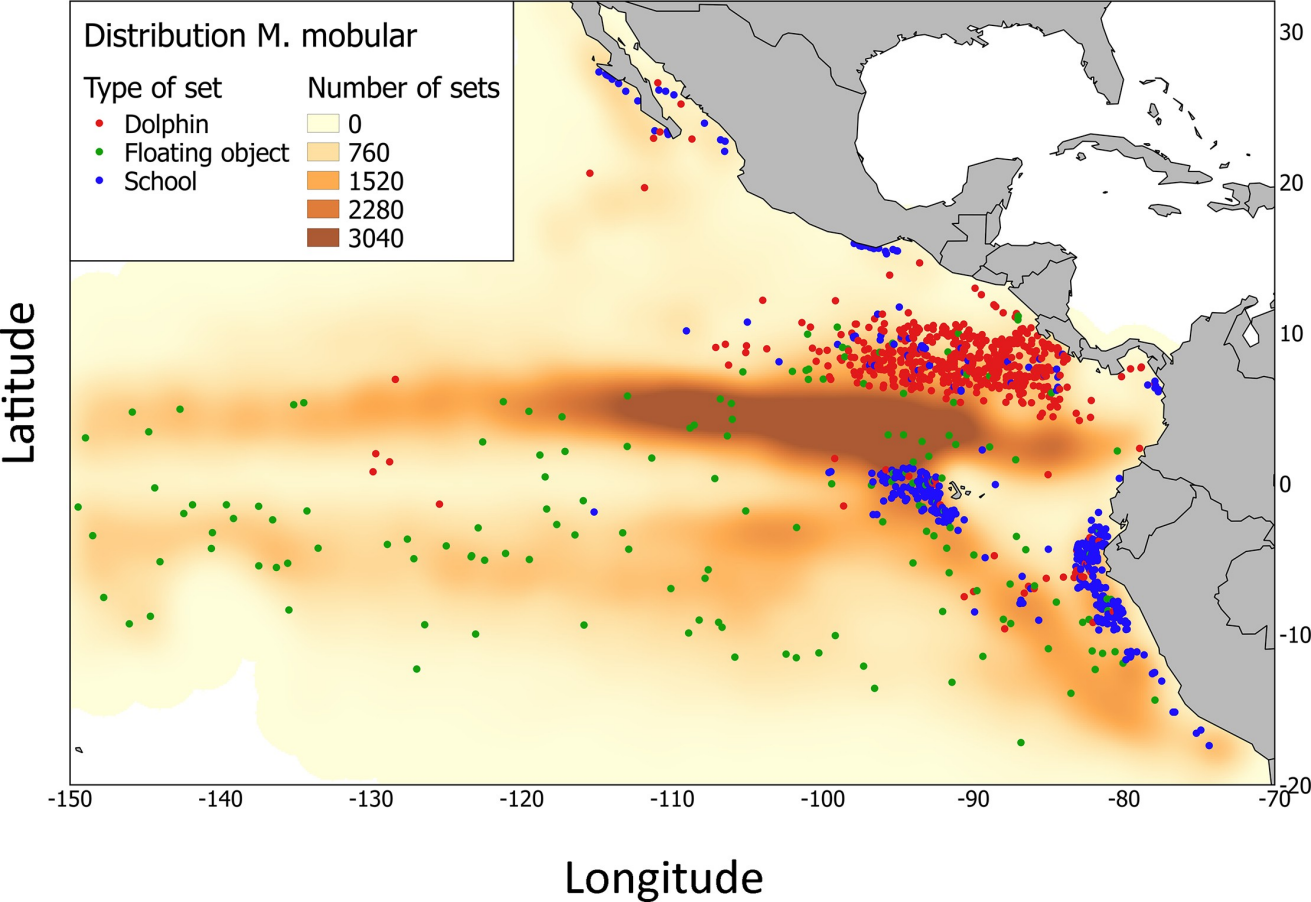
Distribution of sets with presence of *Mobula mobular* for the years 2005–2015 in Dolphin sets (red points), Floating object sets (green points) and School sets (blue points) from the tropical tuna purse-seine fishery in the eastern Pacific Ocean (limited by 30°N, 20°S, 70°W and 150°W). Effort (number of sets) for all bycatches (not only mobulids) represented in orange and created by using kernel density estimation from the function “heatmap” in Quantum GIS, 2014.

**Fig 2:**
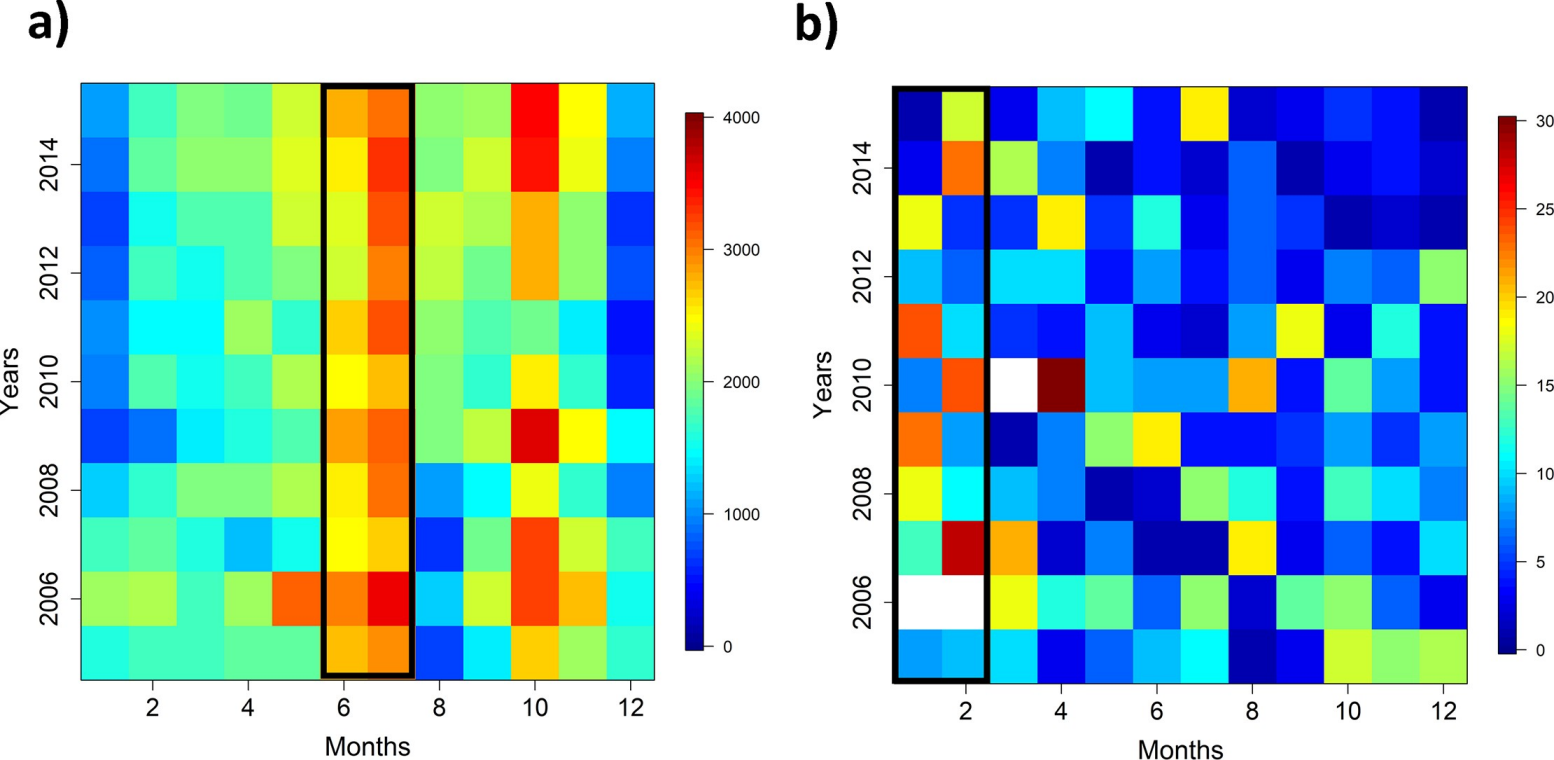
a) Total number of distribution of sets in the fishery represented by years (2005–2015) on the y-axis and months (1–12) on the x-axis. b) Total number of sets with presence of *Mobula mobular* represented by years (2005–2015) and months (1–12) (b).

Out of 261272 catch observations, only 1270 with presence of *Mobula mobular* were found in the study period between 2005–2015. There were 572 (~45.04%) Dolphin, 163 (~12.83%) Floating objects and 535 (~42.13%) School sets with presence of *M*. *mobular* (Fig 1). There were 20727 Dolphin, 224831 Floating objects and 14444 School sets with not presence of the species. While most of the fishing effort (total number of sets) from the bycatch database is located north of the equator, close to the front system, sets with presence of *M*. *mobular* were found in different locations, such as Galapagos or the Costa Rica Dome, dominated by Dolphin and School sets. The highest number of total sets (sets with presence and no presence of *M*. *mobular*) per month (1420) was made during July (Fig 2). In contrast, the highest number of sets with presence of *M*. *mobular* per month was found during the northern winter (January-February) (203 and 197; respectively) (Fig 2).

### Generalized additive model

After studying all the possible combinations of covariates (13 candidate models), the final model (based on the lowest AIC value; S2 Table) included as explanatory variables a) spatial variables (latitude-longitude interaction), b) temporal variables (month), c) type of fishing mode (set type as factor) and d) environmental variables (oxygen, chlorophyll, nitrate and sea surface height). The more relevant models (13) were presented, as the others that have only one variable of difference presented and similar AIC values.

The final model explained 31.3% of the total deviance with an adjusted r^2^ of 0.31 (Table 2). The individual contribution of the variables is shown in Table 2. The most important covariates to explain the presence of *M*. *mobular* are the type of set (21.6%), followed by the interaction latitude-longitude (18.4%), chlorophyll (7.36%) and sea surface height (7.28%).

**Table 2:** Summary results for the final Generalized Additive Model (GAM) selected to model the presence of *Mobula mobular* in the eastern Pacific Ocean (2005–2015). Individual contribution of each variable (% Deviance) running the model separately. SSH (sea surface height), O2 (oxygen), Ni (nitrate).

Family	Binomial		
Link function	Log		
Adjusted R2	0.31		
Deviance explained	31.3%		
	e.d.f	p-value	% Deviance
Latitude * Longitude	18.638	< 2e-16	18.40%
Type	-4.3937	<2e-16	21.60%
Month	3.64	2.47E-05	2.71%
Chlorophyll	2.99	7.23E-10	7.36%
SSH	2.111	3.11E-07	7.28%
O2	1.559	1.88E-05	1.11%
Ni	2.009	1.47E-07	0.86%

*interaction

**Fig 3:**
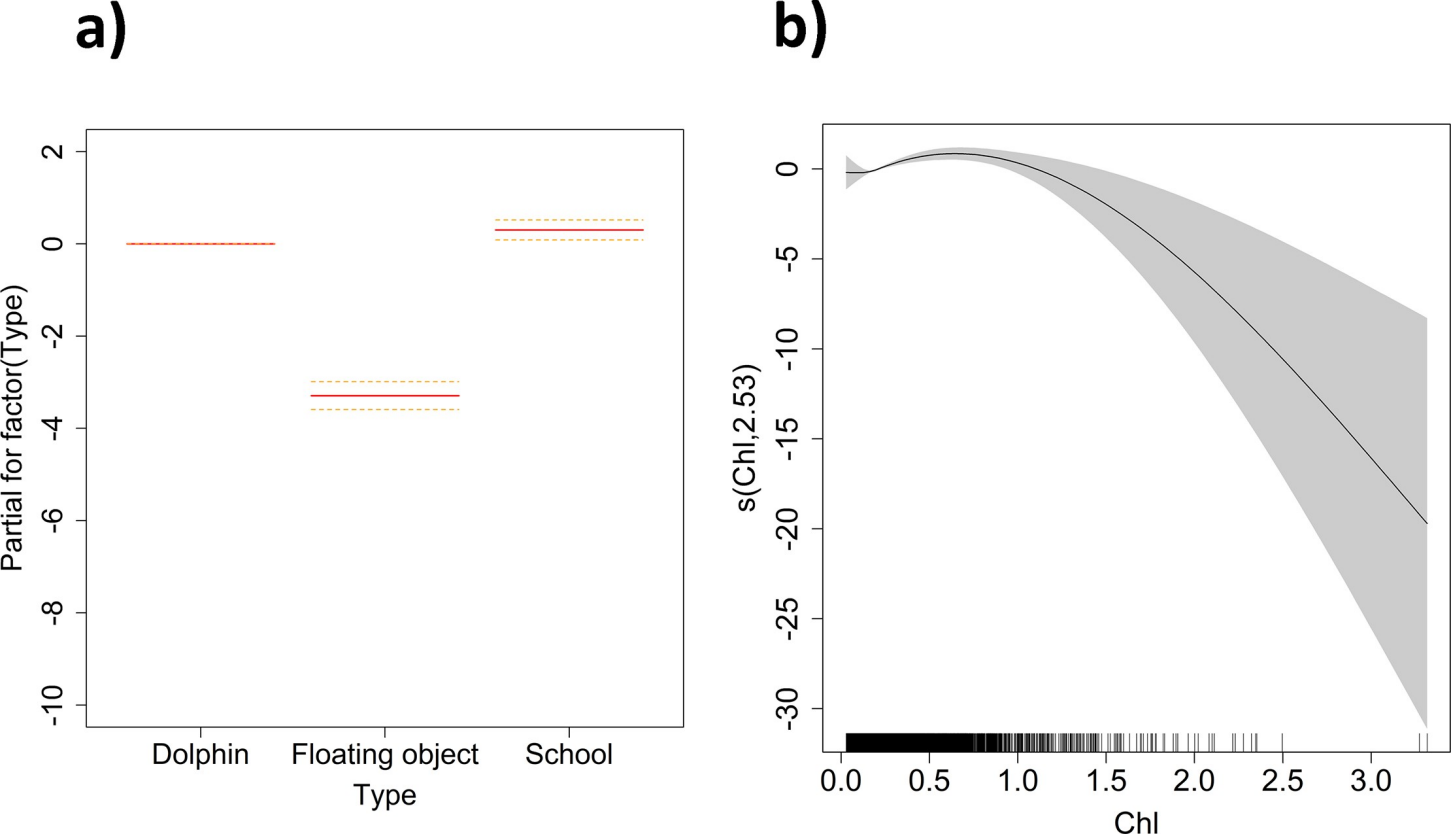
**Smoothed fits of covariates modeling the presence of *Mobula mobular* for: a) Type of set (Dolphin vs. Floating object vs. School) and Chl (chlorophyll, in mg·m**^**-3**^ **in x-axis) variables.** The y-axis represents the spline function. Shaded polygons indicate approximate 95% confidence bounds.

**Fig 4:**
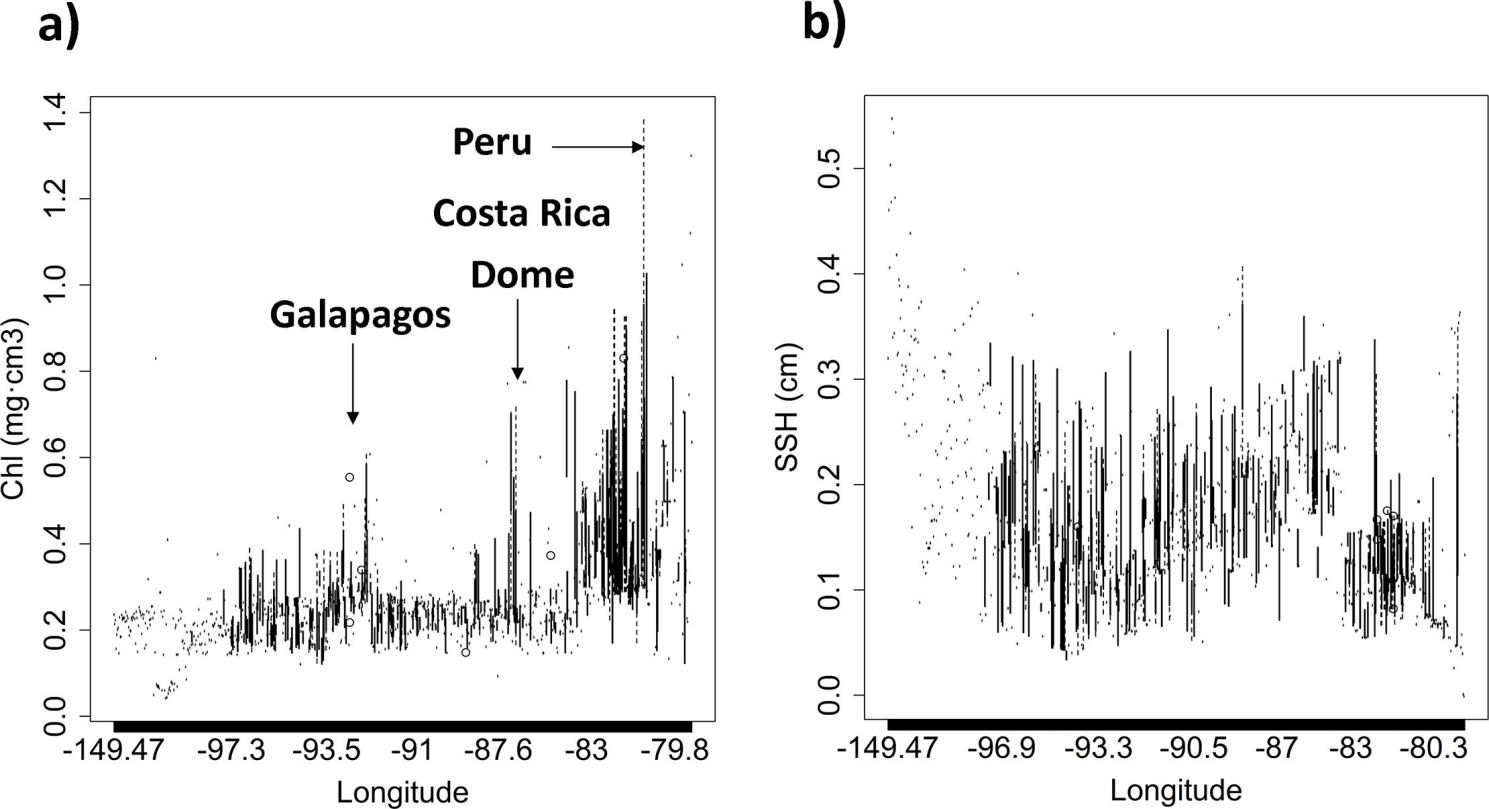
Lineplots showing the sets (longitude values in x-axis) with presence of *Mobula mobular* and their associated a) Chlorophyll concentrations (Chl, in y-axis) and b) Sea surface height (SSH, in y-axis) values.

The smooth functions of the response variable for each of the covariates considered in the model are shown in Fig 3 and S3 Fig. The model suggests higher presence of *M*. *mobular* in Dolphin and School sets in areas close to the coast (80–100°W) between 0°N and 20°S during December-January, and to a lesser extent, during August-September. The significant interaction of latitude-longitude suggests presence of *Mobula mobular* close to the coast with three main hotspots predicted: off the Peru coast, the Galapagos Islands and the Costa Rica Dome (S3 Fig). With respect to the oceanographic variables, *M*. *mobular* is likely to be found more often in areas with low concentration of oxygen (< 200 mg/l), relatively high concentration of chlorophyll (0.5–1 mg·m^-3^), low sea surface height values (close to 0 cm) and medium concentration of nitrate (around 9 mg/l) (Fig 3 and S3 Fig). Lineplots in Fig 4 showed the longitudinal distribution of the chlorophyll and sea surface height for the sets with presence of *M*. *mobular*. The three peaks of chlorophyll identified in Fig 4 correspond to the areas around Costa Rica Dome (87°W), Galapagos (92.4°W) and Peru (81°W). In the case of the sea surface height, values close to 0 were found in coastal areas (80–82°W) and close to Galapagos (94°W), directly related to eddy circulation and upwelling systems (Fig 4).

The ability of the model to predict *M*. *mobular* presence was good, especially given the very low number of presences in the data set (Sensitivity: 0.44). The TSS indicated a correlation between the predicted presence of *M*. *mobular* and the observed presence, with a TSS value of 0.42. In addition, the AUC for the model was 0.92 and the specificity was 0.97 (Table 3).

**Table 3:** Accuracy indices to evaluate the performance of the model for each interaction and the mean: Area Under the Curve (AUC), True Skill Statistic (TSS), Sensitivity and Specificity.

Interaction	AUC	TSS	Sensitivity	Specificity
1	0.92	0.51	0.55	0.96
2	0.94	0.51	0.54	0.97
3	0.91	0.44	0.47	0.97
4	0.91	0.30	0.32	0.98
5	0.91	0.32	0.34	0.98
**Mean**	0.92	0.42	0.44	0.97

The spatial autocorrelation of the residuals from the best final model was also explored. Results from the Moran test indicated that there was no significant spatial autocorrelation in the residuals (Moran’s I = 0.99).

**Fig 5:**
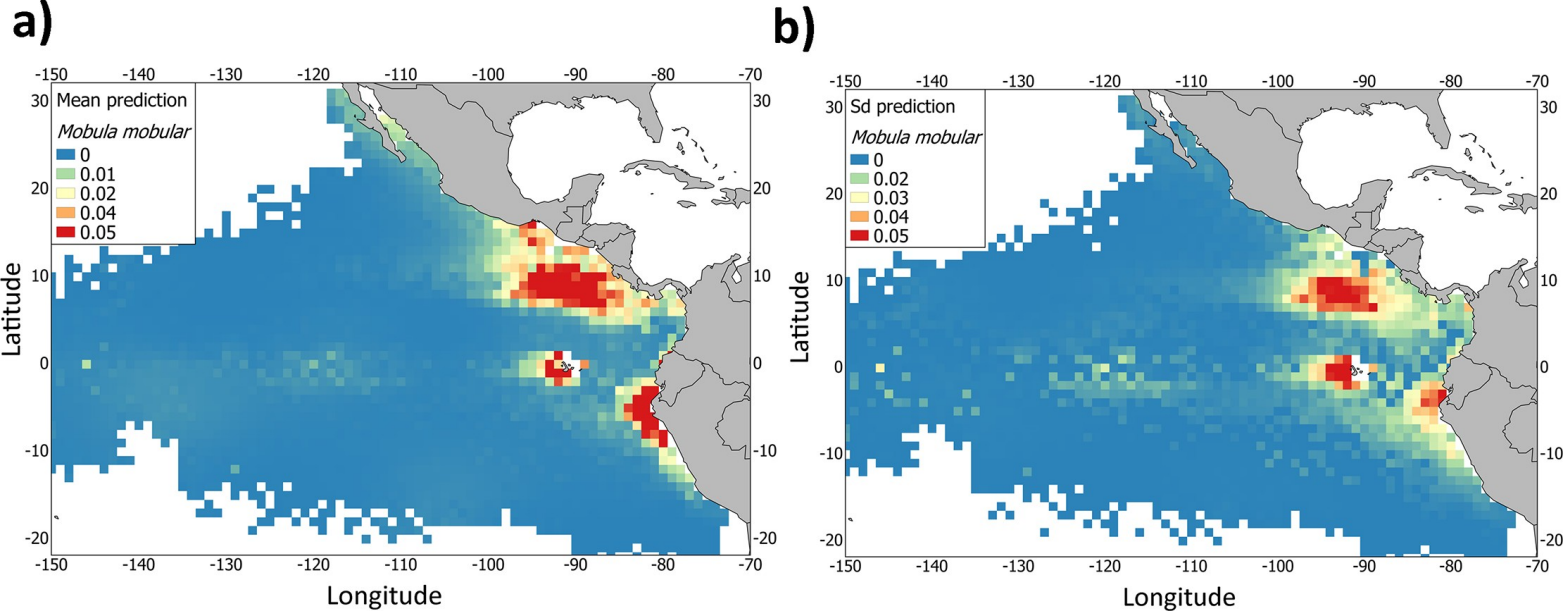
Mean prediction and standard deviation of the presence of *Mobula mobular* bycatch from the tropical tuna purse-seine fishery (2005–2015) in the eastern Pacific Ocean based on the most parsimonious model fit.

The prediction maps showed three main hotspots with presence of *M*. *mobular*: i) the area close to the coastal upwelling of Peru, ii) the area west of Galapagos and iii) the area close to the Costa Rica Dome and the coastal upwelling systems of Tehuantepec, Papagayo and Panama (Fig 5). A fourth area, with lower but consistent prediction values (presences persistent at mean, with low standard deviation values), was also identified around of the Baja California Peninsula and the Equatorial area.

**Fig 6:**
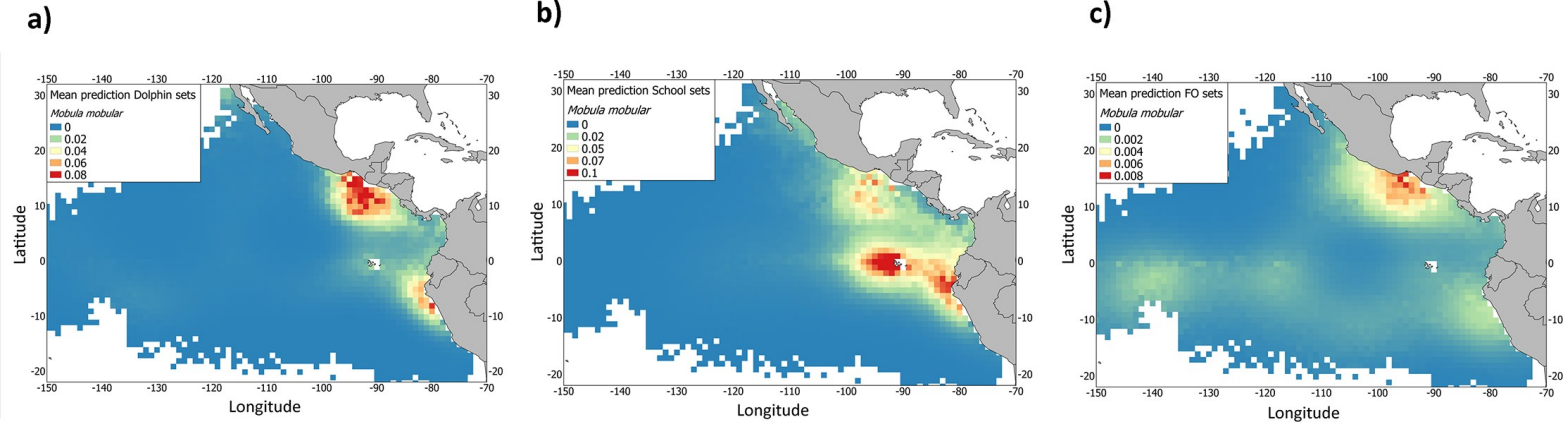
Mean prediction of the presence of *Mobula mobular* bycatch species from the tropical tuna purse-seine fishery (2005–2015) in the eastern Pacific Ocean in a) Dolphin sets, b) School sets and c) Floating object sets based on the most parsimonious model fit.

These areas are distinguished when conducting predictions by set type (Fig 6). The presence of *M*. *mobular* is predicted around the Costa Rica Dome for Dolphin sets (maximum values around 0.08) and around Galapagos, Peru and the coastal upwelling systems for School sets (maximum values around 0.1). In contrast, a presence area around the equator extending from the coast of Peru west to Galapagos was predicted for Floating objects sets, although this area had lower predicted values (maximum values around 0.008) than School and Dolphin sets in the Costa Rica Dome, Galapagos, and coastal upwelling areas.

**Fig 7:**
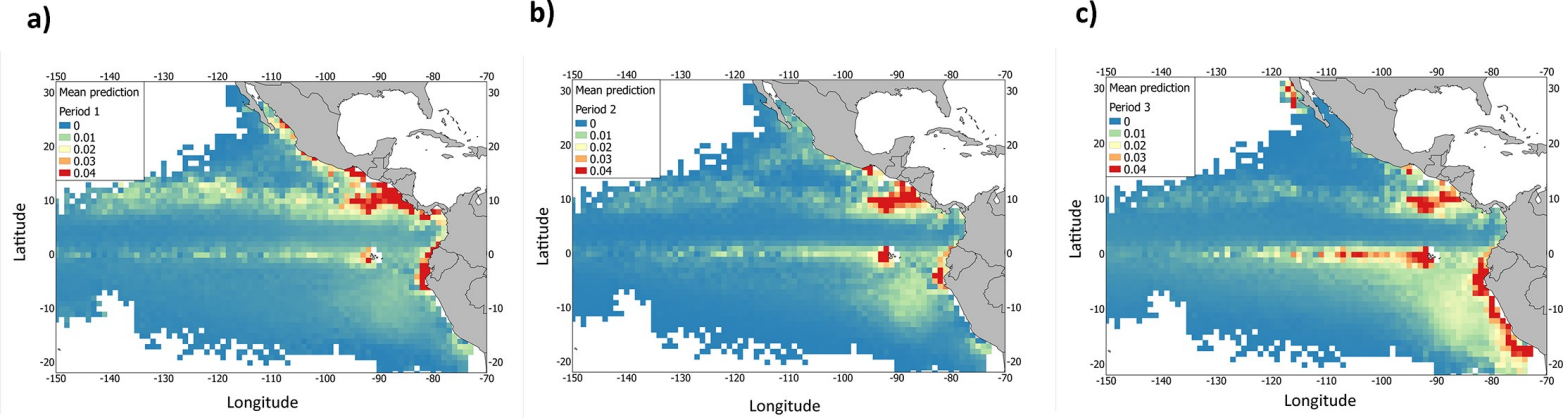
Mean prediction of the presence of *Mobula mobular* bycatch species from the tropical tuna purse-seine fishery (2005–2015) in the eastern Pacific Ocean in a) Period 1, b) Period 2 and c) Period 3 based on the most parsimonious model fit.

Predictions by periods indicated that the presence of *M*. *mobular* could vary seasonally explained by the seasonal upwelling events of the eastern Pacific (Fig 7). The first period considered (February-May) predicted probability of presence of the species around the coast and during the spring upwelling events in Central America. The second period predicted presence of *M*. *mobular* around the Costa Rica Dome and the third one, around Peru, Galapagos, the Pacific coast of the Gulf of California and the equatorial area.

## Discussion

The identification of the main areas of distribution for pelagic species is always a challenge due to the difficulties of obtaining extensive spatial and temporal sampling coverage in their ocean pelagic environment. In this study, GAMs were used to identify the major environmental factors that affect the occurrence of *Mobula mobular* bycatch in purse-seine sets using fishery-dependent data from the IATTC observer program.

Results of this study suggest that *M*. *mobular* distribution is directly related to seasonal upwelling systems with high productivity in the eastern Pacific Ocean. Understanding the spatial distribution of this species is essential for the protection of their populations.

### Environmental and spatial-temporal characteristics of *M*. *mobular*

Habitat preferences of marine species can be described according to one or more physical or chemical variables such as temperature, chlorophyll, salinity or oxygen content. Those variables directly characterize particular water masses [76].

We identified chlorophyll (Chl) (7.36% of deviance explained) and sea surface height (SSH) (7.28%) as the main environmental variables that explain the presenceof *M*. *mobular* in the tropical tuna purse-seine fishery in the eastern Pacific Ocean. This suggests that the seasonal distribution of the species is primarily related to variations in chlorophyll concentrations and sea surface height values in relation to upwelling systems. Specifically, we observed higher probability of presence of *M*. *mobular* in waters with relatively high concentration of chlorophyll (0.5–1 mg·m^-3^). These values (with fluctuations depending on the season) of chlorophyll can be found in the most important upwelling areas of the eastern Pacific Ocean, such as the Galapagos Islands, the upwelling system off Peru, the costal upwelling system of the Gulf of Tehuantepec, Papagayo and Panama, the Costa Rica Dome and the Gulf of California [56, 57]. The decline of the probability of presence of *M*. *mobular* at higher concentrations of chlorophyll (Fig 3) may be explained by the data limitation at those levels. Similar ranges of chlorophyll have been described as preferred for manta ray species in other locations around the world [1, 44, 45, 49, 52, 77]. However, in the case of *M*. *mobular* and other devil rays, most studies have been based on tagging data, including depth and sea surface temperature as explanatory variables for distribution and movements, but not chlorophyll. In that sense, this study provides new information about the distributional preferences of the species related to chlorophyll concentrations. In addition, it suggests that mobulids most likely universally target high-productivity regions that provide ideal foraging opportunities. In the case of sea surface height, *M*. *mobular* was more likely to be present in areas with low sea surface height values. This result may reveal a preference of the species for cyclonic eddies, upwelling systems, shallow mixed layers or cold sides of thermal fronts [25]. Similar relationships (positive or negatives) in association with SSH values have been also found for other bycatch species, such as turtles [34], mahi-mahis [25], wahoos [78] and sailfishes [26].

The lack of significance of Year (considered as a factor) in the model indicates that there is not annual trend with respect to the presence in the bycatch. This fact could be probably explained by the fact that (1) there is not enough information in our dataset to find differences between years, and/or 2) because the studied species have a similar spatial distribution every year but probably with different annual abundances that should be studied in future works. In any case, differences between years should be explorer deeper to find possible relationships between important events of El Niño and La Niña.

Sea surface temperature (SST) was not included in the final model, since it was correlated with oxygen and with sea surface height. Despite sea surface temperature (SST) can directly describe the presence of upwelling systems (observed by low temperature values), in this study SST was not considered in the final model (i.e. not significant) which was replaced by other covariables, such as oxygen (O2) and sea surface height (SSH). In the case of O2, it was included in the model because mobulid rays are normally found at the surface and in shallow waters with low concentration of O2. Thus, the distribution of these species is totally conditioned by the bathymetry of the area and the concentration of oxygen at these shallow waters that may induce easier captures by the fisheries. Understanding the preference of the species by the concentrations of oxygen, we could know if there is more probability of finding the species in other studied area. In the case of SSH, this variable can be an indicator of presence of the species in areas where the convergences and divergences (upwellings and downwellings) are important. Therefore, both variables, O2 and SSH combined with chlorophyll provide more specific information about the relationship of the species with the upwelling systems than those just described by the SST; which could be more occasional.

In the case of the oxygen and nitrate, the contribution of these two variables to the model was minimal. However, they showed values that correspond with typical upwelling systems’ characteristics [56, 79]. Since the oxygen determines the vertical distribution of mobulid rays, SST and SSH could explain the presence of the species in upwelling systems. In the case of nitrate, surface waters in areas of upwelling systems contain similar values of nitrate as the areas where more probability of presence of our species was found; leading to correlate again these upwelling areas with the habitat of *M*. *mobular*. The inclusion of both variables improved the model fits and contributed to our understanding of environmental preferences of the species. Thus, should be considered in future SDMs for mobulid species in addition to other covariates such as temperature and chlorophyll. The relationship between mobulid distribution and oxygen minimum zones should be studied in greater detail (e.g. Stewart et al., 2018), especially in areas with shoaling oxygen minimum zones such as the Gulf of California or the Pacific Warm Pool area [80, 81].

Presence of *M*. *mobular* was explained not only by environmental variables but also by spatial-temporal variables of the purse seine fishery and their types of sets. Our model revealed that the interaction latitude-longitude was very important variable. The inclusion of this interaction allowed accounting for the spatial prediction of the species in regions such as the Costa Rica Dome, the Galapagos Islands and the coast of Peru. These areas correspond with important areas of productivity and therefore, of great importance for the ecology of the species. Lezama Ochoa (13) reported high number of mobulids bycatch in the Costa Rica Dome region but also some bycatch in coastal areas between 1993 and 2014. These areas are considered representative regions of biological hotspots [57]. The Costa Rica Dome could play an important role in the distribution of *M*. *mobular*; as mobulids seem to aggregate in this area only during a short period (July and August) [13] (S1 Fig). The persistent pattern of cyclonic wind stress curl lifts the thermocline, creating a center of oceanic upwelling and leading to elevated chlorophyll concentrations, low mean sea level anomaly values, and high biological production [56, 57, 82]. This area concentrates nutrients and influences the abundance and distribution not only of mobulids, but also of other marine species, such as sharks, billfishes, tunas, marine mammals or turtles [8, 38, 57, 83, 84]. In winter, prior to the formation of the Costa Rica Dome, wind jets blowing through Central America and Mexico generate the persistent formation of eddies with high chlorophyll concentration in the Gulfs of Tehuantepec and Papagayo [57]. Finally, in the case of Galapagos and Peru, the biological richness of their waters are the result of two upwelling systems. Both upwellings register highest regional concentrations of chlorophyll [57] making the coast off Peru and Galapagos very important habitats for mobulids and whale sharks [85, 86].

With respect to set type, the highest presence of *M*. *mobular* was predicted in School sets, followed by Dolphin sets, while the prediction in Floating object sets was much lower [9, 13]. This could be the case because the different purse seiner fishery modes operates in different areas which varied productivity regimes and, hence, differential mobulids preferential habitat. For example, School sets followed large yellowfin tuna schools which are mostly located in productivity areas foraging in lower prey of the food chain. Therefore, the probability of presence of *M*. *mobular* seems to be set type-specific. Regions with different probability of presence of the species were observed depending of the fishing mode: Peru, Galapagos and the Gulf of California were the main areas with presence of *M*. *mobular* in School sets, the Costa Rica Dome in Dolphin sets and the Equatorial area in Floating object sets [13]. Presences in the Costa Rica Dome are mainly in Dolphin sets, which are the primary set type used in the region. The coast of Peru and Galapagos are the secondary hotspots for *M*. *mobular* captures, which occur mainly in School sets in these areas. The different fishing sets could be indicative of different schooling behavior depending on different oceanographic conditions. That could result in indications of regional preferential environmental conditions of *M*. *mobular* linked to similar oceanographic conditions of the tuna schools associated to different type of sets. Similarly, other species of devil ray have been observed feeding on baitfish alongside dolphins in the eastern Pacific [87]. These suggest that the presence of *M*. *mobular* in School and Dolphin sets on tunas could be a result of foraging on zooplankton in high-productivity regions where tunas are also likely to be found. In contrast, *M*. *mobular* was notably rare in Floating object sets, suggesting that they are not generally attracted to FADs (unlike tunas or sharks), and instead target higher productivity regions to feed, as reflected in the model results. This conclusion is supported by the prediction maps by set type (Fig 6), as well as by the results from the model. Despite Floating object sets exhibiting the highest bycatches for other species group in the eastern Pacific Ocean tuna purse-seine fishery [26, 88], our model accounted for a very low overall mobulid bycatch for this type of set. That could be related to the lack of overlap between the spatial dynamic of floating object fishery and mobulids preferential habitat.

With respect to temporal variability, the model predicted higher presence of the species during late northern winter (December-January) and summer (August). Previous studies [19] established that these seasonal distributions could be related to food availability. For example, as filter feeders, whale sharks can be attracted to high productivity systems that create increased zooplankton biomass [89–91]. Similarly, mobulid rays’ spatial distributions and aggregations are thought to be explained by the seasonal distribution of prey species [22, 45, 92–94]. Studies reporting on the stomach contents, and carbon (ᵹ^13^C) and nitrogen (ᵹ^15^N) stable isotope characteristics of mobulid species have shown direct relationships between their spatial distribution and food availability in tropical and subtropical areas. This is because most of the diet of mobulids made up of zooplankton and occasionally small fishes [22, 53, 87, 95, 96]. For example, *Mobula thurstoni* has been primarily observed feeding on euphausiids [97], but also on mysid shrimps [22]; while *Mobula munkiana* has been observed feeding on the mysid *Mysidium sp*. [22, 98]. Rohner, Burgess [16] found seasonal distribution patterns of mobulids species in the Bohol Sea related with feeding habits on the euphausids krill *Euphausia diomedeae* during six months (November to May). This species of krill was recorded in 91% of the stomachs of *Mobula japanica* (now *M*. *mobular*) and *M*. *thurstoni*, while squid and fishes were also found in *Mobula tarapacana’s* stomach content and myctophid fishes and copepods in *Mobula birostris* stomachs. With respect to *M*. *japanica*, Masangcay, Metillo [99] established its diet using data on its stomach contents and stable isotopes in Philippines. The authors concluded that most of the stomach content of *M*. *japanica* was composed of the tropical krill *Pseudeuphausia latifrons* during January to May, which coincides with the season when upwelling occurs throughout the area, and therefore, when the krill is most abundant.

In the case of the Pacific Ocean, most of the studies of prey preference of *M*. *japanica* based on its stomach contents are limited to the Gulf of California [22, 98] where the euphasiid *Nyctiphanes simplex* appears to be the main prey of *M*. *mobular* [22, 98]. Notarbartolo-di-Sciara [22] found that 99.6% of the stomach content of *M*. *mobular* was made up of *N*. *simplex*, and that *M*. *mobular* sightings seemed to coincide with periods of high *N*. *simplex* biomass. Our modeled distribution patterns support the hypothesis that the presence of *M*. *mobular* is directly related with areas and seasons of food availability, represented by areas of high concentration of chlorophyll (considered a proxy of food availability) in upwelling systems during winter and during summer. In the case of the Gulf of California, presence of *M*. *mobular* was predicted for this area by our model, but was not identified as one of the main areas of importance in this study. This is because the fishing activity and catches of tuna by purse seiners are not large in the Gulf of California and, thus, the model is not predicting a large hotspot due to limited presence data in the region. Therefore, based on the limited data available of *M*. *mobular* from the bycatch database in the Gulf of California, our model results do not necessarily reflect the true relative abundance of *M*. *mobular* as compared with other regions such as Peru, Galapagos, and the Costa Rica Dome.

### Future challenges

One of the main challenges in ecology is correctly describing and understanding the processes that determine the distribution of organisms, as these processes are inevitably associated with a degree of uncertainty [100]. Identifying the factors that could cause this uncertainty may help to obtain better future model performance. There is not a single best model for evaluating habitat preferences and distributions of species, and the choice of the model type and the variables selected should be driven by the objective of the study [101].

In this study, Generalized Additive Models allowed us to predict the distribution of *Mobula mobular* bycatch species in the eastern Pacific Ocean with good predictive power. However, the use of bycatch data from a fishery that is focused on catching tuna and not mobulids leads to limitations in how the results can be interpreted [34]. This is particularly true for the areas where the fleet does not operate or partially operates, such as the Gulf of California. In the case for other areas, where the effort is important the results could be considered sound. To account for these potential biases in the fishery-dependent data source, future studies should attempt to include additional data sources such as acoustic and/or satellite tag data or fisheries-independent surveys for validation of the models.

The quality of model outputs depends greatly on the input variables (biological data and environmental data) [102]. The presence of *M*. *mobular* was modeled instead of the abundance in order to better characterize the fundamental relationships between species distribution and environmental variables. However, other techniques (such as Maximum Entropy Models, Bayesian Approaches, Random Forests, etc.) should be applied in future studies to model the presence but also the seasonal abundance of the species. Additionally, the inclusion of zooplankton data could improve the model performance and help to identify seasonal patterns of *M*. *mobular* in areas of high zooplankton abundance. Specifically, data on *Nictiphanex simplex* biomass, which is the main prey of *M*. *mobular* based on studies conducted in the eastern Pacific, could be included in future habitat and distribution models. Unfortunately, the bycatch monitoring program and fisheries databases do not typically collect this type of data, so other sources should be explored or future data collection efforts undertaken [34].

### Conservation challenges

For any effective fishery management regulation, a good knowledge of the habitat of the species is essential to minimize the interaction of the fisheries with the most vulnerable species [32]. Fishery-dependent data can provide a long time-series, wide spatial coverage all year-round when long-term monitoring data over board geographical ranges are limited [103]. Yet little is known about the biology and distribution of mobulid ray species, especially because they are difficult to study in the wide oceanic habitat, even if they are aggregated in specific regions [104]. In that sense, SDMs, based on fishery observer programs, could be used to provide essential knowledge about their spatial distribution and to identify biological hotspots and the ocean environment that characterizes them [32, 57]. This knowledge will be fundamental to develop effective spatial fishery management regulations which could halt the reduction of vulnerable populations such as mobulids.

Hotspots could vary temporally and spatially, for example those related to mesoscale processes (i.e. fronts and eddies), but they can also be related to physical features that persist through the year. Both type of hotspots are ecologically very relevant; which could be used to implement spatial (adaptive) management plans [57]. For example, regions of high productivity, where mobulids and tunas could show variable degree of distribution overlap depending of the time of year [57]. Some productive areas, such as north off Peru or Galapagos, show important *M*. *mobular* hotspot totally overlap with the main fishing ground of tropical tuna purse seine at the same time (e.g. from December to February); which difficult the application of possible management measures. In contrast, consistent (year by year) high aggregations of mobulids have been found during late July-early August in the Costa Rica Dome [13]. Despite the fishing effort (in Dolphin sets) is not low in this area during this period, possible management options could cover a shorter and narrower period compared with the areas of Peru and Galapagos. At the same time, the fishing effort (mainly in Dolphin sets) is also important on other areas of the Convention area (such as north of equator between 10–25° N), during this period; where mobulid species seem not to be aggregated (see S4 Fig). The location of the Dome changes over the year [56] and presents important oceanographic characteristics (high concentration of chlorophyll, low values of oxygen) which attract large concentration of marine species from zooplankton to top predators [79]; including high aggregations of *M*. *mobular* during summer. Understanding the position and extent of the Dome would be, therefore, of great relevance for the management and conservation of this species in this area. Future studies should address the habitat use and the spatial distribution of the different mobulid species in this area, as well as the study of the possible effect of environmental changes (El Niño or climate change) on their distributions. The identification and description of dynamic habitats (based on dynamic maps of biological hotspots) like the Costa Rica Dome could allow to develop habitat-specific (adaptive) management strategies based on natural system variations.

There has been a notably increase in the number on mobulid species publications (concerning photo-ID studies, aerial surveys, bycatch and tagging studies). There are major knowledge gaps yet in areas such as taxonomy, life history (age and growth and mortality), population trends, movements or spatial dynamics [6, 10], which are critical for performing stocks assessments. There are usually few species-specific datasets of fishery statistics and biological data available to develop robust stock assessment models to support the development of appropriate management measures [105]. Therefore, alternative methods are required in such data-limited situations that can provide fishery managers with sufficient information to prioritize potential species of concern. IATTC [106] employed Ecological Risk Assessment (ERA) and specifically, Productivity-Susceptibility Analysis (PSA), as an alternative assessment approach to determine the relative vulnerability of data-limited bycatch species impacted by fisheries in the EPO, including mobulid rays. However, for developing correct PSA analyses good knowledge of the distribution of the species is required. In that sense, the species predicted distribution maps provided by the SDMs could fill this knowledge gap. More recently, Griffiths, Kesner-Reyes [107] developed a flexible quantitative approach that uses less input parameters than PSA to quantify the cumulative impacts of multiple fisheries on data-poor bycatch species. Application of this new approach to EPO industrial longline and purse-seine fisheries to a range of species group with varying life histories, including *Mobula mobular*, is currently in progress. Until these new methods can be fully implemented to advice in specific management measures, measures such as spatial management measures are required to ensure mobulid’s survival. For that, SDMs could be used to identify specific-spatial management measures to mitigate the mortality of the species with the least possible impact on fishing operations.

Based on the old bad practices applied to release mobulid rays from the purse-seiners [14], the IATTC implemented a resolution (C-15-04) to prohibit retaining mobulids onboard and to apply new release handling techniques. As most of the mobulid rays caught by the purse seiner fishery are considered mortalities by the IATTC even if they are released, the reduction of post-release mortality of mobulid species in the tropical tuna purse-seine fishery is a challenge for their conservation in the eastern Pacific Ocean [10]. To date, the factors driving the changes in mobulid ray populations have not been well understood, but it is believed that the modification in the handling behavior of the fishermen could contribute to halt the population declines and ensure their survival [5, 13, 108]. Francis and Jones [20] found that the handling techniques used after capture mobulids may strongly influence their post-release survival. Therefore, there is a need to find handling best practice to increase their post-release survival, as well as to evaluate the post-release mortality of mobulid capture incidentally in the purse-seine vessels [10]. New handling practices developed in the purse seiners fishery [108] showed very encouraging results, with significant reductions in capture mortality when good practices are implemented (M. A. Hall, personal communication); which should be extended to all oceans where tropical tuna purse seiners are operating.

Finally, and despite the actions by some countries (Australia, Brazil, the Member States of the European Union, Israel, Mexico, Ecuador, New Zealand, and the Maldives) to offer increase protection for mobulid species impacted by fisheries [5], other threats, such as habitat destruction, pollution, unregulated tourism or climate change are still affecting their populations. Therefore, for the implementation of successful measures to reduce the mortality of these species in purse seiner fisheries, it is necessary the involvement of all stakeholders (the scientists, industry, governments and the ONGs) in the discussions and decision making process to achieve the common goal of conserving these iconic species.

## Conclusions

This study improves our understanding of the spatial, temporal and environmental preferences of *Mobula mobular* in the tropical eastern Pacific Ocean based on purse-seine fishery-dependent data using Generalized Additive Models (GAMs). The model results suggested that *M*. *mobular* distribution is directly related to highly productive oceanographic features that occur in the eastern Pacific Ocean, such as the Peru and Galapagos upwelling systems and the Costa Rica Dome. Chlorophyll and sea surface height were the main oceanographic variables that explained the distribution of the species in the study area, and Dolphin and School sets dominated the mobulid presence events while fewer probability of presences occurred in Floating object sets. The results also showed that the Costa Rica Dome could be considered as an area of interest for mobulids conservation. This information is crucial to identify the dynamic of mobulids habitats that could be managed and protected under dynamic spatial management measures to reduce the mortality of mobulid rays in the eastern Pacific Ocean and, hence, ensure the sustainability of the populations of these iconic species.

## Supporting information

S1 Fig Major geographic and oceanographic features of the Eastern Tropical Pacific Ocean: California and Peru Currents, Eastern Pacific Warm Pool, upwelling systems of the Gulf of Tehuantepec, Papagayo and Panama, the Costa Rica Dome and the Equatorial Cold Tongue. SeaWiFS mean monthly chlorophyll (mg·m^−3^) concentrations in a) January and b) August for years 2000–2015. Source: http://oceandata.sci.gsfc.nasa.gov/.
(DOCX)

S2 Fig Study of correlation and collinearity between variables by calculating Pearson’s rank correlation and the Variance Inflation Factor (VIF). (DOCX)

S3 Fig Smoothed fits of covariates modeling the presence of *Mobula mobular* for: Interaction of latitude and longitude (Lat*Lon), Type of set (Dolphin vs. Floating object vs. School), Month, O2 (oxygen, in mg/l in x-axis), Ni (nitrate, in mg/l in x-axis), SSH (sea surface height, in cm in x-axis), and Chl (chlorophyll, in mg·m^-3^ in x-axis) variables. The y-axis represents the spline function. Shaded polygons indicate approximate 95% confidence bounds.
(DOCX)

S4 Fig Distribution of number of sets (n) and total number of individuals of *Mobula mobular* in 5x5 green squares in Dolphin sets for July and August. Dark green represents squares where highest number of individuals were observed (*created by Marlon Román*, *IATTC*).
(DOCX)

S1 Table a,b. Example of script used to download environmental variables using Phyton and R routine code.
(DOCX)

S2 Table Final candidate Generalized Additive Models (GAMs) with the corresponded Akaike Information Criteria (AIC) values and the variables selected for each model (up). The piece-wise construction of the best model with each new variable improving the AIC value (below).
(DOCX)

## References

[1] Jaine F, Couturier L, Weeks S, Townsend K, Bennett M, Fiora K, et al When giants turn up: sighting trends, environmental influences and habitat use of the manta ray Manta alfredi at a coral reef. PloS one. 2012;7(10):e46170-e. 10.1371/journal.pone.0046170 23056255 PMC3463571

[2] Deakos MH, Baker JD, Bejder L. Characteristics of a manta ray Manta alfredi population off Maui, Hawaii, and implications for management. Marine Ecology Progress Series. 2011;429:245–60.

[3] O'Malley Mp, Townsend KA, Hilton P, Heinrichs S, Stewart JD. Characterization of the trade in manta and devil ray gill plates in China and South‐east Asia through trader surveys. Aquatic Conservation: Marine and Freshwater Ecosystems. 2017;27(2):394–413.

[4] Heinrichs S, O'Malley M, Medd H, Hilton P. Manta ray of hope: global threat to manta and mobula rays Manta Ray of Hope Project (https://www.mantarayofhope.com). 2011.

[5] Lawson JM, Fordham SV, O’Malley MP, Davidson LN, Walls RH, Heupel MR, et al Sympathy for the devil: a conservation strategy for devil and manta rays. PeerJ. 2017;5:e3027 10.7717/peerj.3027 28316882 PMC5354073

[6] Couturier L, Marshall A, Jaine F, Kashiwagi T, Pierce S, Townsend K, et al Biology, ecology and conservation of the Mobulidae. Journal of fish biology. 2012;80(5):1075–119. 10.1111/j.1095-8649.2012.03264.x 22497374

[7] Acebes JMV, Tull M. The history and characteristics of the mobulid ray fishery in the Bohol Sea, Philippines. PloS one. 2016;11(8):e0161444 10.1371/journal.pone.0161444 27575536 PMC5004919

[8] Croll DA, Dewar H, Dulvy NK, Fernando D, Francis MP, Galván‐Magaña F, et al Vulnerabilities and fisheries impacts: the uncertain future of manta and devil rays. Aquatic conservation: marine and freshwater ecosystems. 2016.

[9] Hall MA, Roman M. Bycatch and non-tuna catch in the tropical tuna purse seine fisheries of the world. FAO fisheries and aquaculture technical paper. 2013;568.

[10] Stewart JD, Jaine FR, Armstrong AJ, Armstrong AO, Bennett MB, Burgess KB, et al Research priorities to support effective manta and devil ray conservation. Frontiers in Marine Science. 2018.

[11] Mas F, Forselledo R, Domingo A. Mobulid ray by-catch in longline fisheries in the south-western Atlantic Ocean. Marine and Freshwater Research. 2015;66(9):767–77.

[12] Dulvy NK, Fowler SL, Musick JA, Cavanagh RD, Kyne PM, Harrison LR, et al Extinction risk and conservation of the world’s sharks and rays. Elife. 2014;3. 10.7554/eLife.00590 PMC3897121 24448405

[13] Lezama Ochoa NH, Martin; Roman Marlon; Vogel Nick. Spatial and temporal distribution of mobulid ray species in the eastern Pacific Ocean ascertained from observer data from the tropical tuna purse-seine fishery. Environmental biology of fishes. 2018.

[14] Poisson F, Séret B, Vernet A-L, Goujon M, Dagorn L. Collaborative research: Development of a manual on elasmobranch handling and release best practices in tropical tuna purse-seine fisheries. Marine Policy. 2014;44:312–20.

[15] Pardo SA, Kindsvater HK, Cuevas-Zimbrón E, Sosa-Nishizaki O, Pérez-Jiménez JC, Dulvy NK. Growth, productivity, and relative extinction risk of a data-sparse devil ray. Scientific reports. 2016;6:33745 10.1038/srep33745 27658342 PMC5034314

[16] Rohner CA, Burgess KB, Rambahiniarison JM, Stewart JD, Ponzo A, Richardson AJ. Mobulid rays feed on euphausiids in the Bohol Sea. Royal Society Open Science. 2017;4(5):161060 10.1098/rsos.161060 28572998 PMC5451799

[17] IATTC. Recommendations by the staff for conservation measures in the eastern Pacific Ocean. Document IATTC 89-04d. 89th Meeting Guayaquil, Ecuador. Inter-American Tropical Tuna commission 2015.

[18] White WT, Corrigan S, Yang L, Henderson AC, Bazinet AL, Swofford DL, et al Phylogeny of the manta and devilrays (Chondrichthyes: mobulidae), with an updated taxonomic arrangement for the family. Zoological Journal of the Linnean Society. 2017;182(1):50–75.

[19] Croll DA, Newton KM, Weng K, Galván-Magaña F, Sullivan JO, Dewar H. Movement and habitat use by the spine-tail devil ray in the Eastern Pacific Ocean. Marine ecology progress series. 2012;465:193–200.

[20] Francis MP, Jones EG. Movement, depth distribution and survival of spinetail devilrays (Mobula japanica) tagged and released from purse‐seine catches in New Zealand. Aquatic conservation: marine and freshwater ecosystems. 2016.

[21] Anderson RC, Adam MS, Goes JI. From monsoons to mantas: seasonal distribution of Manta alfredi in the Maldives. Fisheries Oceanography. 2011;20(2):104–13.

[22] Notarbartolo-di-Sciara G. Natural history of the rays of the genus Mobula in the Gulf of California. Fishery Bulletin. 1988;86(1):45–66.

[23] Elith J, Leathwick JR. Species Distribution Models: Ecological Explanation and Prediction Across Space and Time. Annual Review of Ecology, Evolution, and Systematics. 2009;40:677–97. 10.1146/annurev.ecolsys.110308.120159 PubMed PMID: 4249*.

[24] Brodie S, Hobday AJ, Smith JA, Everett JD, Taylor MD, Gray CA, et al Modelling the oceanic habitats of two pelagic species using recreational fisheries data. Fisheries oceanography. 2015;24(5):463–77.

[25] Marín‐Enríquez E, Seoane J, Muhlia‐Melo A. Environmental modeling of occurrence of dolphinfish (Coryphaena spp.) in the Pacific Ocean off Mexico reveals seasonality in abundance, hot spots and migration patterns. Fisheries Oceanography. 2018.

[26] Martinez-Rincon RO, Ortega-Garcia S, Vaca-Rodriguez JG, Griffiths SP. Development of habitat prediction models to reduce by-catch of sailfish (Istiophorus platypterus) within the purse-seine fishery in the eastern Pacific Ocean. Marine and Freshwater Research. 2015;66(7):644–53.

[27] Venables WN, Dichmont CM. GLMs, GAMs and GLMMs: an overview of theory for applications in fisheries research. Fisheries Research. 2004;70(2):319–37.

[28] Bigelow K, Musyl MK, Poisson F, Kleiber P. Pelagic longline gear depth and shoaling. Fisheries research. 2006;77(2):173–83.

[29] Perryman HA, Babcock EA. GENERALIZED ADDITIVE MODELS FOR PREDICTING THE SPATIAL DISTRIBUTION OF BILLFISHES AND TUNAS ACROSS THE GULF OF MEXICO. Collect Vol Sci Pap ICCAT. 2017;73(5):1778–95.

[30] Eveson JP, Patterson TA, Hartog JR, Evans K. Modelling surfacing behaviour of southern bluefin tuna in the Great Australian Bight. Deep Sea Research Part II: Topical Studies in Oceanography. 2018.

[31] Carvalho FC, Murie DJ, Hazin FH, Hazin HG, Leite-Mourato B, Burgess GH. Spatial predictions of blue shark (Prionace glauca) catch rate and catch probability of juveniles in the Southwest Atlantic. ICES Journal of Marine Science. 2011;68(5):890–900.

[32] Hahlbeck N, Scales KL, Dewar H, Maxwell SM, Bograd SJ, Hazen EL. Oceanographic determinants of ocean sunfish (Mola mola) and bluefin tuna (Thunnus orientalis) bycatch patterns in the California large mesh drift gillnet fishery. Fisheries Research. 2017;191:154–63.

[33] Escalle L, Pennino MG, Gaertner D, Chavance P, Delgado de Molina A, Demarcq H, et al Environmental factors and megafauna spatio‐temporal co‐occurrence with purse‐seine fisheries. Fisheries oceanography. 2016;25(4):433–47.

[34] Montero JT, Martinez‐Rincon RO, Heppell SS, Hall M, Ewal M. Characterizing environmental and spatial variables associated with the incidental catch of olive ridley (Lepidochelys olivacea) in the Eastern Tropical Pacific purse‐seine fishery. Fisheries oceanography. 2016;25(1):1–14.

[35] Martínez Rincón RO. Efecto de la variabilidad ambiental en la distribución de las capturas incidentales de pelágicos mayores en el Océano Pacífico Oriental [Doctoral dissertation]: Instituto Politécnico Nacional. Centro Interdisciplinario de Ciencias Marinas; 2012.

[36] Martínez-Rincón RO, Ortega-García S, Vaca-Rodriguez JG. Incidental catch of dolphinfish (C*oryphaena* spp.) reported by the Mexican tuna purse seiners in the eastern Pacific Ocean. Fisheries Research. 2009;96(2):296–302.

[37] Lezama-Ochoa N, Murua H, Chust G, Ruiz J, Chavance P, de Molina AD, et al Biodiversity in the by-catch communities of the pelagic ecosystem in the Western Indian Ocean. Biodiversity and conservation. 2015;24(11):2647–71.

[38] Lezama-Ochoa N, Murua H, Hall M, Román M, Ruiz J, Vogel N, et al Biodiversity and habitat characteristics of the by-catch assemblages in Fish Aggregating Devices (FADs) and Free School sets in the Eastern Pacific Ocean. Frontiers in Marine Science. 2017.

[39] Lopez J, Moreno G, Lennert-Cody C, Maunder M, Sancristobal I, Caballero A, et al Environmental preferences of tuna and non-tuna species associated with drifting fish aggregating devices (DFADs) in the Atlantic Ocean, ascertained through fishers’ echo-sounder buoys. Deep Sea Research Part II: Topical Studies in Oceanography. 2017;140:127–38.

[40] Lezama‐Ochoa N, Murua H, Ruiz J, Chavance P, Delgado de Molina A, Caballero A, et al Biodiversity and environmental characteristics of the bycatch assemblages from the tropical tuna purse seine fisheries in the eastern Atlantic Ocean. Marine Ecology. 2018:e12504.

[41] Notarbartolo-di-Sciara G, Hillyer EV. Mobulid rays off eastern Venezuela (Chondrichthyes, Mobulidae). Copeia. 1989:607–14.

[42] Fortuna CM, Kell L, Holcer D, Canese S, Filidei E Jr, Mackelworth P, et al Summer distribution and abundance of the giant devil ray (Mobula mobular) in the Adriatic Sea: baseline data for an iterative management framework. Scientia Marina. 2014;78(2):227–37.

[43] di Sciara GN, Lauriano G, Pierantonio N, Cañadas A, Donovan G, Panigada S. The Devil We Don't Know: Investigating Habitat and Abundance of Endangered Giant Devil Rays in the North-Western Mediterranean Sea. PloS one. 2015;10(11):e0141189 10.1371/journal.pone.0141189 26580814 PMC4651356

[44] Girondot M, Bédel S, Delmoitiez L, Russo M, Chevalier J, Guéry L, et al Spatio-temporal distribution of Manta birostris in French Guiana waters. Journal of the Marine Biological Association of the United Kingdom. 2015;95(1):153–60.

[45] Hacohen‐Domené A, Martínez‐Rincón RO, Galván‐Magaña F, Cárdenas‐Palomo N, Herrera‐Silveira J. Environmental factors influencing aggregation of manta rays (Manta birostris) off the northeastern coast of the Yucatan Peninsula. Marine Ecology. 2017;38(3):e12432.

[46] Duffy C, Abbott D. Sightings of mobulid rays from northern New Zealand, with confirmation of the occurrence of Manta birostris in New Zealand waters. 2003.

[47] Clark TB. Abundance, home range, and movement patterns of manta rays (Manta alfredi, M. birostris) in Hawaiʻi: [Honolulu]:[University of Hawaii at Manoa],[12 2010]; 2010.

[48] Canese S, Cardinali A, Romeo T, Giusti M, Salvati E, Angiolillo M, et al Diving behavior of the giant devil ray in the Mediterranean Sea. Endangered Species Research. 2011;14(2):171–6.

[49] Graham RT, Witt MJ, Castellanos DW, Remolina F, Maxwell S, Godley BJ, et al Satellite tracking of manta rays highlights challenges to their conservation. PloS one. 2012;7(5):e36834 10.1371/journal.pone.0036834 22590622 PMC3349638

[50] Braun CD, Skomal GB, Thorrold SR, Berumen ML. Movements of the reef manta ray (Manta alfredi) in the Red Sea using satellite and acoustic telemetry. Marine biology. 2015;162(12):0.

[51] Braun CD, Skomal GB, Thorrold SR, Berumen ML. Diving behavior of the reef manta ray links coral reefs with adjacent deep pelagic habitats. PloS one. 2014;9(2):e88170 10.1371/journal.pone.0088170 24516605 PMC3916408

[52] Jaine F, Rohner C, Weeks S, Couturier L, Bennett M, Townsend KA, et al Movements and habitat use of reef manta rays off eastern Australia: offshore excursions, deep diving and eddy affinity revealed by satellite telemetry. Marine Ecology Progress Series. 2014;510:73–86.

[53] Stewart JD, Hoyos-Padilla EM, Kumli KR, Rubin RD. Deep-water feeding and behavioral plasticity in Manta birostris revealed by archival tags and submersible observations. Zoology. 2016;119(5):406–13. 10.1016/j.zool.2016.05.010 27461910

[54] Thorrold SR, Afonso P, Fontes J, Braun CD, Santos RS, Skomal GB, et al Extreme diving behaviour in devil rays links surface waters and the deep ocean. Nature Communications. 2014;5:4274 10.1038/ncomms5274 24983949 PMC4102113

[55] Lezama-Ochoa N, Hall M, Murua H, Newton K, Croll D. Habitat model to predict the spatial distribution of Mobula japanica bycatch species in the eastern Pacific. 2018, in preparation.

[56] Fiedler PC, Lavín MF. Oceanographic conditions of the Eastern Tropical Pacific Coral Reefs of the Eastern Tropical Pacific: Springer; 2017 p. 59–83.

[57] Palacios DM, Bograd SJ, Foley DG, Schwing FB. Oceanographic characteristics of biological hot spots in the North Pacific: a remote sensing perspective. Deep Sea Research Part II: Topical Studies in Oceanography. 2006;53(3):250–69.

[58] Team RC. R: A language and environment for statistical computing. R Foundation for Statistical Computing, Vienna, Austria 2016. 2017.

[59] Hijmans RJ, van Etten J. raster: Geographic analysis and modeling with raster data. R package version. 2012;1:9–92.

[60] Guisan A, Edwards TC Jr, Hastie T. Generalized linear and generalized additive models in studies of species distributions: setting the scene. Ecological modelling. 2002;157(2):89–100.

[61] Giannoulaki M, Iglesias M, Tugores MP, Bonanno A, Patti B, De Felice A, et al Characterizing the potential habitat of European anchovy Engraulis encrasicolus in the Mediterranean Sea, at different life stages. Fisheries Oceanography. 2013;22(2):69–89.

[62] Jones A, Hosegood P, Wynn R, De Boer M, Butler-Cowdry S, Embling C. Fine-scale hydrodynamics influence the spatio-temporal distribution of harbour porpoises at a coastal hotspot. Progress in Oceanography. 2014;128:30–48.

[63] Wood S. mgcv 1.3. R package. http://cran.r-project.org. 2006.

[64] Lopez J, Alvarez-Berastegui D, Soto M, Murua H. Modelling the oceanic habitats of silky shark (Carcharhinus falciformis). Implications for conservation and management. 2017.

[65] Wood S. Generalized additive models: an introduction with R: CRC press; 2006.

[66] Zuur A, Ieno E, Walker N, Saveliev A, Smith G. Mixed effects models and extensions in ecology with R Gail M, Krickeberg K, Samet JM, Tsiatis A, Wong W, editors. New York, NY: Spring Science and Business Media 2009.

[67] Akaike H. A new look at the statistical model identification. Automatic Control, IEEE Transactions on. 1974;19(6):716–23.

[68] Ortega-García S, Camacho-Bareño E, Martínez-Rincón RO. Effects of environmental factors on the spatio-temporal distribution of striped marlin catch rates off Cabo San Lucas, Baja California Sur, Mexico. Fisheries Research. 2015;166:47–58.

[69] Kohavi R, editor A study of cross-validation and bootstrap for accuracy estimation and model selection. IJCAI; 1995.

[70] Pearson RG. Species’ distribution modeling for conservation educators and practitioners. Synthesis American Museum of Natural History. 2007;50.

[71] Elith J, Graham CH, Anderson RP, Dudík M, Ferrier S, Guisan A, et al Novel methods improve prediction of species' distributions from occurrence data. Ecography. 2006:129–51.

[72] Fielding AH, Bell JF. A review of methods for the assessment of prediction errors in conservation presence/absence models. Environmental conservation. 1997;24(01):38–49.

[73] Freeman E, Freeman ME. Package ‘PresenceAbsence’. R Package Version. 2012;1(9).

[74] Bivand R, Anselin L, Assunçaõ R, Berke O, Bernat A, Carvalho M, et al spdep: Spatial dependence: Weighting schemes, statistics and models. R package version 0.4–24. 2009.

[75] Wood S, Wood MS. The mgcv package. https://www.r-project.org 2007.

[76] Hobday A, Young J, Moeseneder C, Dambacher J. Defining dynamic pelagic habitats in oceanic waters off eastern Australia. Deep Sea Research Part II: Topical Studies in Oceanography. 2011;58(5):734–45.

[77] Martínez Urrea DA. Influencia de factores ambientales sobre la distribución de la manta gigante (Manta birostris) en Holbox, Quintana Roo: Instituto Politécnico Nacional Centro Interdisciplinario de Ciencias Marinas; 2015.

[78] Martínez-Rincón RO, Ortega-García S, Vaca-Rodríguez JG. Comparative performance of generalized additive models and boosted regression trees for statistical modeling of incidental catch of wahoo (Acanthocybium solandri) in the Mexican tuna purse-seine fishery. Ecological Modelling. 2012;233:20–5.

[79] Jiménez JA. The Thermal Dome of Costa Rica: An oasis of productivity off the Pacific Coast of Central America. MarViva Foundation, San José, Costa Rica 2017:106 pp.

[80] Stramma L, Prince ED, Schmidtko S, Luo J, Hoolihan JP, Visbeck M, et al Expansion of oxygen minimum zones may reduce available habitat for tropical pelagic fishes. Nature Climate Change. 2012;2(1):33–7.

[81] Gilly WF, Beman JM, Litvin SY, Robison BH. Oceanographic and biological effects of shoaling of the oxygen minimum zone. Annual review of marine science. 2013;5:393–420. 10.1146/annurev-marine-120710-100849 22809177

[82] Kessler WS. The circulation of the eastern tropical Pacific: A review. Progress in Oceanography. 2006;69(2):181–217.

[83] Ballance LT, Pitman RL, Fiedler PC. Oceanographic influences on seabirds and cetaceans of the eastern tropical Pacific: a review. Progress in Oceanography. 2006;69(2):360–90.

[84] Shillinger GL, Swithenbank AM, Bograd SJ, Bailey H, Castelton MR, Wallace BP, et al Identification of high-use internesting habitats for eastern Pacific leatherback turtles: role of the environment and implications for conservation. Endangered Species Research. 2010;10:215–32.

[85] Hearn AR, Acuna D, Ketchum JT, Penaherrera C, Green J, Marshall A, et al Elasmobranchs of the Galapagos marine reserve The Galapagos Marine Reserve: Springer; 2014 p. 23–59.

[86] Chavez FP, Messié M. A comparison of eastern boundary upwelling ecosystems. Progress in Oceanography. 2009;83(1):80–96.

[87] Stewart JD, Barroso A, Butler RH, Munns RJ. Caught at the surface: myctophids make easy prey for dolphins and devil rays. Ecology. 2018. 10.1002/ecy.2348 29870592

[88] Hall MA, Alverson DL, Metuzals KI. By-catch: problems and solutions. Marine Pollution Bulletin. 2000;41(1):204–19.

[89] Hacohen-Domené A, Galvan-Magaña F, Ketchum-Mejia J. Abundance of whale shark (Rhincodon typus) preferred prey species in the southern Gulf of California, Mexico. Cybium. 2006;30(4):99–102.

[90] Hacohen-Domené A, Martínez-Rincón RO, Galván-Magaña F, Cárdenas-Palomo N, de la Parra-Venegas R, Galván-Pastoriza B, et al Habitat suitability and environmental factors affecting whale shark (Rhincodon typus) aggregations in the Mexican Caribbean. Environmental biology of fishes. 2015:1–12.

[91] Heyman WD, Graham RT, Kjerfve B, Johannes RE. Whale sharks Rhincodon typus aggregate to feed on fish spawn in Belize. Marine Ecology Progress Series. 2001;215:275–82.

[92] Stewart JD, Rohner CA, Araujo G, Avila J, Fernando D, Forsberg K, et al Trophic overlap in mobulid rays: insights from stable isotope analysis. Marine Ecology Progress Series. 2017;580:131–51.

[93] Celona A, editor Caught and observed giant devil rays Mobula mobular (Bonnaterre, 1788) in the Strait of Messina. Annales Ser hist nat; 2004.

[94] Borrell A, Cardona L, Kumarran RP, Aguilar A. Trophic ecology of elasmobranchs caught off Gujarat, India, as inferred from stable isotopes. ICES Journal of Marine Science. 2010;68(3):547–54.

[95] Rohner CA, Flam AL, Pierce SJ, Marshall AD. Steep declines in sightings of manta rays and devilrays (Mobulidae) in southern Mozambique. PeerJ Preprints, 2017 2167–9843.

[96] Burgess K. Feeding ecology and habitat use of the giant manta ray Manta birostris at a key aggregation site off mainland Ecuador. 2017.

[97] Gadig OBF, Namora RC, dos Santos Motta F. Occurrence of the bentfin devil ray, Mobula thurstoni (Chondrichthyes: Mobulidae), in the western Atlantic. Journal of the Marine Biological Association of the United Kingdom. 2003;83(4):869–70.

[98] Sampson L, Galván-Magaña F, De Silva-Dávila R, Aguíñiga-García S, O'Sullivan JB. Diet and trophic position of the devil rays Mobula thurstoni and Mobula japanica as inferred from stable isotope analysis. Journal of the Marine Biological Association of the United Kingdom. 2010;90(05):969–76.

[99] Masangcay SIG, Metillo EB, Hayashizaki K-I, Tamada S, Nishida S. Feeding Habits of Mobula japanica (Chondrichthyes, Mobulidae) in Butuan Bay, Mindanao Island, Philippines. Science Diliman. 2018;30(1).

[100] Payne MR, Barange M, Cheung WW, MacKenzie BR, Batchelder HP, Cormon X, et al Uncertainties in projecting climate-change impacts in marine ecosystems. ICES Journal of Marine Science: Journal du Conseil. 2015:fsv231.

[101] Lezama Ochoa N, Murua H, Chust G, Van Loon E, Ruiz J, Hall M, et al Present and future potential habitat distribution of Carcharhinus falciformis and Canthidermis maculata by-catch species in the tropical tuna purse-seine fishery under climate change. Frontiers in Marine Science. 2016;3:34.

[102] Phillips SJ, Dudík M, Elith J, Graham CH, Lehmann A, Leathwick J, et al Sample selection bias and presence-only distribution models: implications for background and pseudo-absence data. Ecological Applications. 2009;19(1):181–97. 19323182 10.1890/07-2153.1

[103] Wang L, Kerr LA, Record NR, Bridger E, Tupper B, Mills KE, et al Modeling marine pelagic fish species spatiotemporal distributions utilizing a maximum entropy approach. Fisheries Oceanography. 2018.

[104] Sobral AFL. Biology, ecology and conservation of Mobulid rays in the Azores 2013.

[105] Griffiths S, Duffy L, Aires-da-Silva A, editors. A preliminary ecological risk assessment of the large-scale tuna longline fishery in the eastern Pacific Ocean using Productivity-Susceptibility Analysis. Document SAC-08-07d IATTC Scientific Advisory Committee Eighth Meeting, La Jolla, California, USA; 2017.

[106] IATTC. Ecosystem Considerations. In Tunas and billfishes in the eastern Pacific Ocean in 2009, Fishery Status Report 8, p. 133–134. Inter-American Tropical Tuna Commission La Jolla. 2010.

[107] Griffiths S, Kesner-Reyes K, Garilao C, Duffy L, Roman M. Development of a flexible Ecological Risk Assessment (ERA) approach for quantifying the cumulative impacts of fisheries on bycatch species in the eastern Pacific Ocean. Inter-American Tropical Tuna Commission Scientific Advisory Committee (Ninth meeting), Document SAC-09-12 2018.

[108] ISSF. Skippers’ Guidebook to sustainable purse seine fishing practices2014. Available from: http://www.issfguidebooks.org/downloadable-guides.

