# Supplementary material for: Environmental characteristics associated with the presence of the Spinetail devil ray (Mobula mobular) in the eastern tropical Pacific
Source: PLoS One. 2019 Aug 7;14(8):e0220854. doi: 10.1371/journal.pone.0220854 (PMC6685623; doi:10.1371/journal.pone.0220854)
Supplement: S1 Fig — SeaWiFS mean monthly chlorophyll (mg·m−3) concentrations in a) January and b) August for years 2000–2015. Source: http://oceandata.sci.gsfc.nasa.gov/. (DOCX) [file pone.0220854.s001.docx]

**S1 Fig. Major geographic and oceanographic features of the Eastern Tropical Pacific Ocean: California and Peru Currents, Eastern Pacific Warm Pool, upwelling systems of the Gulf of Tehuantepec, Papagayo and Panama, the Costa Rica Dome and the Equatorial Cold Tongue. SeaWiFS mean monthly chlorophyll (mg·m^−3^) concentrations in a) January and b) August for years 2000-2015. Source:** [**http://oceandata.sci.gsfc.nasa.gov/**](http://oceandata.sci.gsfc.nasa.gov/)


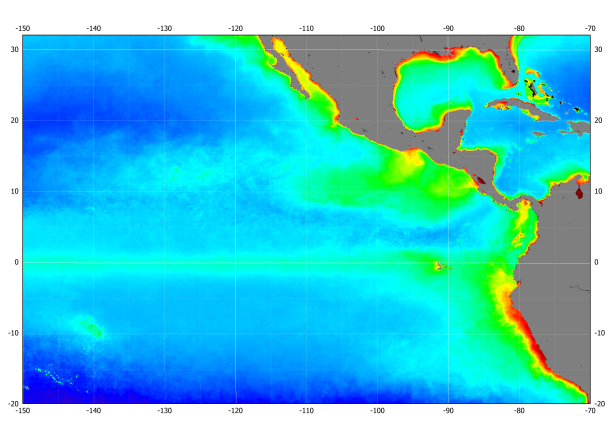

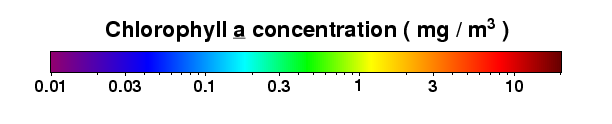

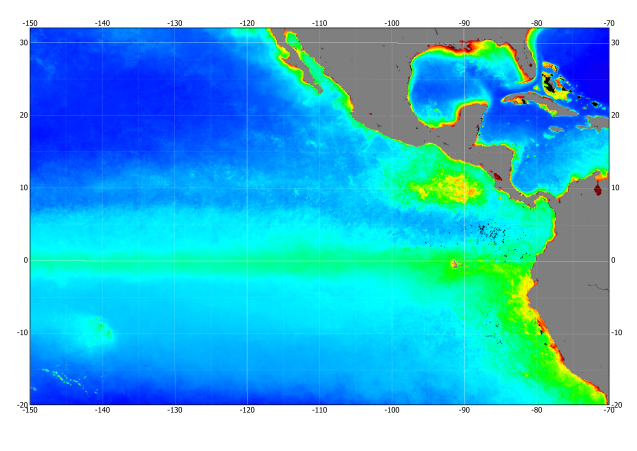

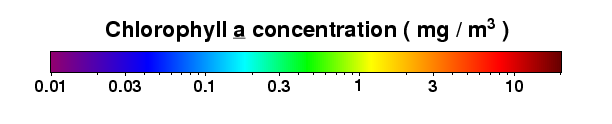


**JANUARY**

**AUGUST**

**Costa Rica Dome**

**Peru**

**Current**

**Gulf of**

**Panama**

**Eastern Pacific Warm Pool**

**Gulf of Tehuantepec**

**Gulf of**

**Papagayo**

California

**Current**

**Equatorial Cold Tongue**
