# Supplementary material for: Environmental characteristics associated with the presence of the Spinetail devil ray (Mobula mobular) in the eastern tropical Pacific
Source: PLoS One. 2019 Aug 7;14(8):e0220854. doi: 10.1371/journal.pone.0220854 (PMC6685623; doi:10.1371/journal.pone.0220854)
Supplement: S2 Fig — (DOCX) [file pone.0220854.s002.docx]

**S2 Fig. Study of correlation and collinearity between variables by calculating Pearson’s rank correlation and the Variance Inflation Factor (VIF)**


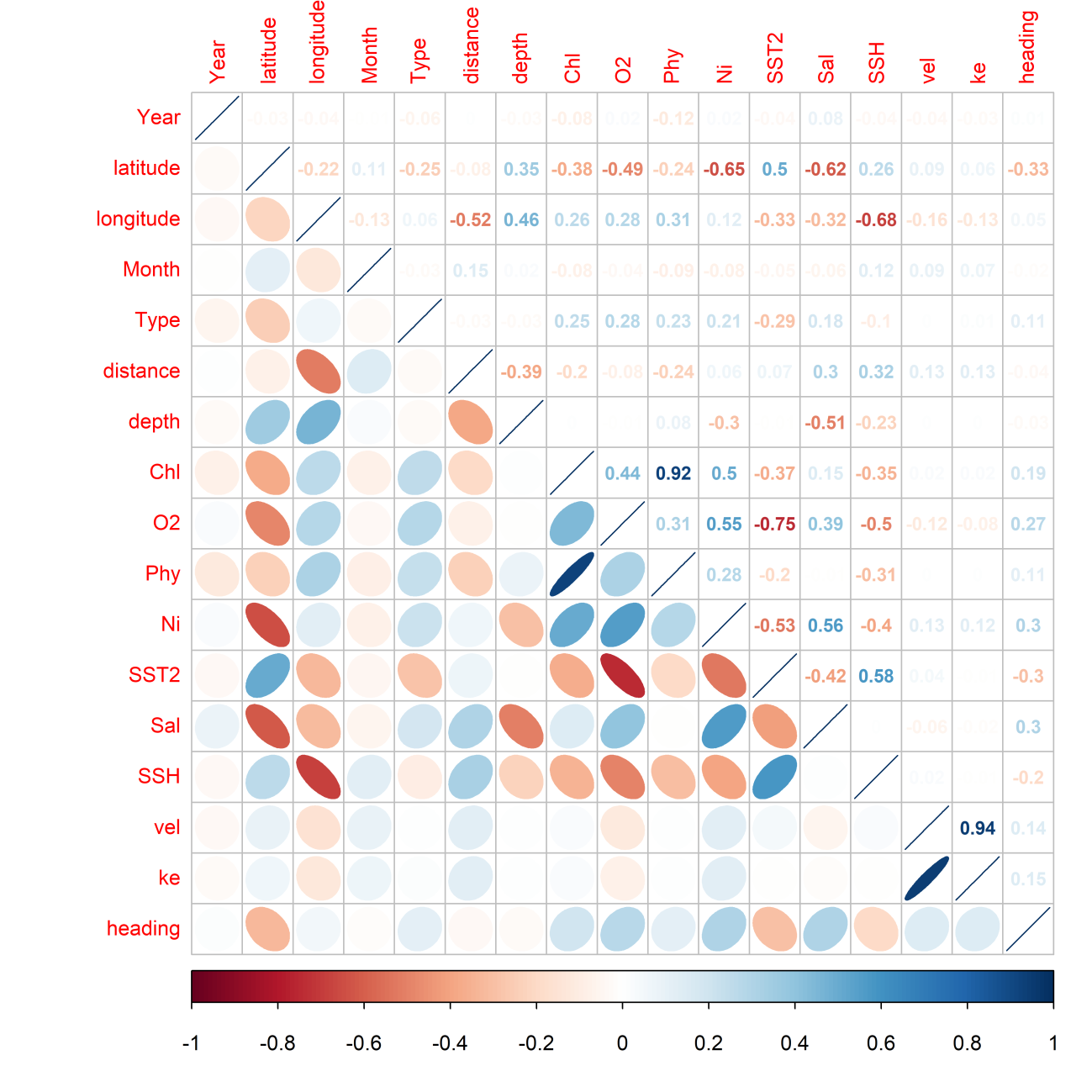


| Variable | GVIF |
| --- | --- |
| Year | 1.040473 |
| latitude | 3.384829 |
| longitude | 4.423902 |
| Month | 1.107073 |
| Type | 1.18712 |
| distance | 1.563855 |
| depth | 1.897131 |
| Chl | 13.057731 |
| O2 | 2.704368 |
| Phy | 10.957683 |
| Ni | 3.475266 |
| SST2 | 3.604257 |
| Sal | 3.559635 |
| SSH | 3.066012 |
| vel | 9.611005 |
| ke | 9.069939 |
| heading | 1.254588 |
