# Supplementary material for: Environmental characteristics associated with the presence of the Spinetail devil ray (Mobula mobular) in the eastern tropical Pacific
Source: PLoS One. 2019 Aug 7;14(8):e0220854. doi: 10.1371/journal.pone.0220854 (PMC6685623; doi:10.1371/journal.pone.0220854)

**S3 Fig. Smoothed fits of covariates modeling the presence of *Mobula mobular* for: Interaction of latitude and longitude (Lat*Lon), Interaction of latitude and longitude three-dimensional, Type of set (Dolphin vs. Floating object vs. School), Month, O2 (oxygen, in mg/l in x-axis), Ni (nitrate, in mg/l in x-axis), SSH (sea surface height, in cm in x-axis), and Chl (chlorophyll, in mg·m^-3^ in x-axis) variables. The y-axis represents the spline function. Shaded polygons indicate approximate 95% confidence bounds.**


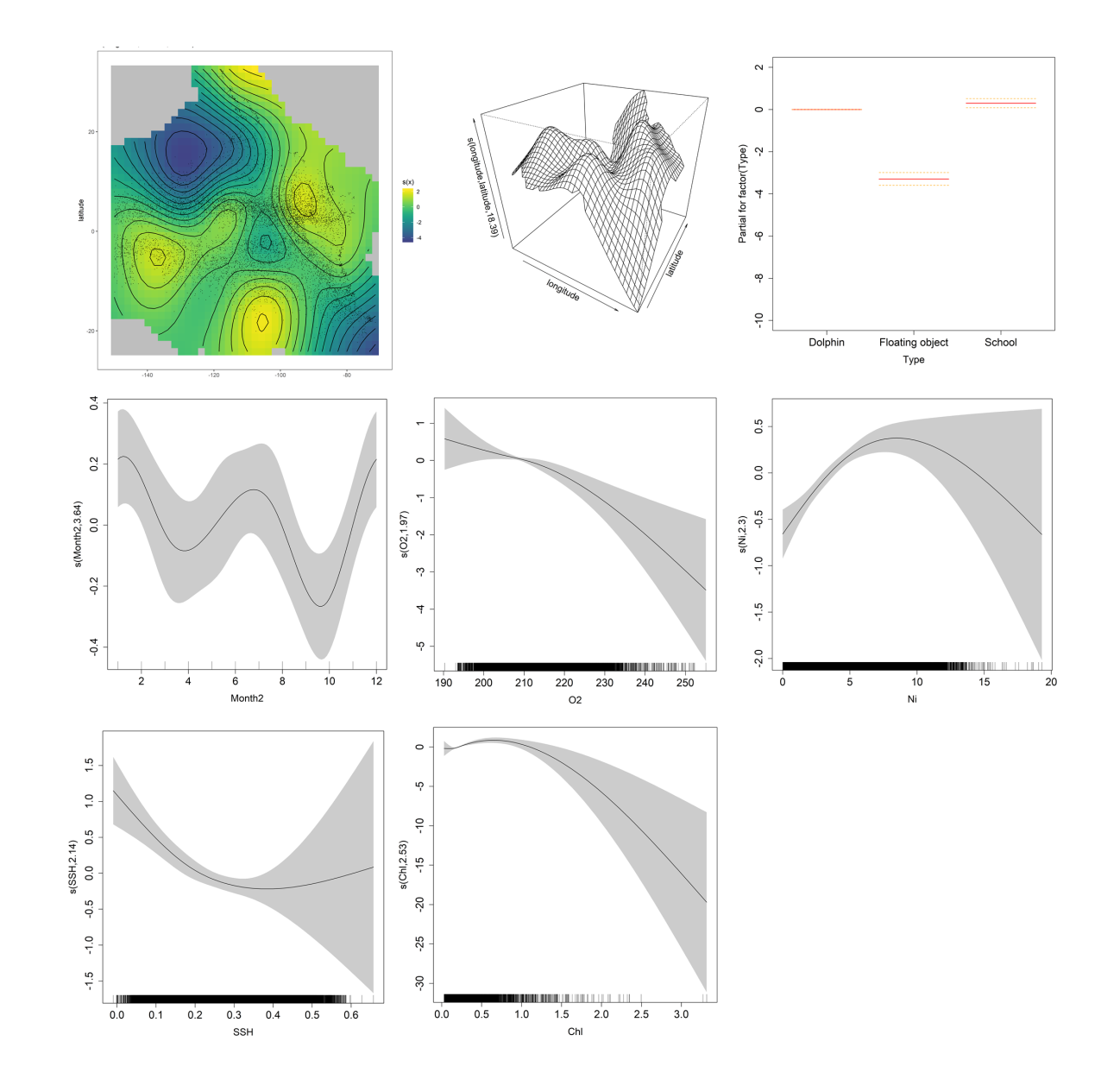

Supplement: S3 Fig — The y-axis represents the spline function. Shaded polygons indicate approximate 95% confidence bounds. (DOCX) [file pone.0220854.s003.docx]
