# Supplementary material for: Environmental characteristics associated with the presence of the Spinetail devil ray (Mobula mobular) in the eastern tropical Pacific
Source: PLoS One. 2019 Aug 7;14(8):e0220854. doi: 10.1371/journal.pone.0220854 (PMC6685623; doi:10.1371/journal.pone.0220854)

**S4 Fig. Distribution of number of sets (n) and total number of individuals of *Mobula mobular* in 5x5 green squares in Dolphin sets for July and August. Dark green represents squares where highest number of individuals were observed (*courtesy by Marlon Román, IATTC*)**


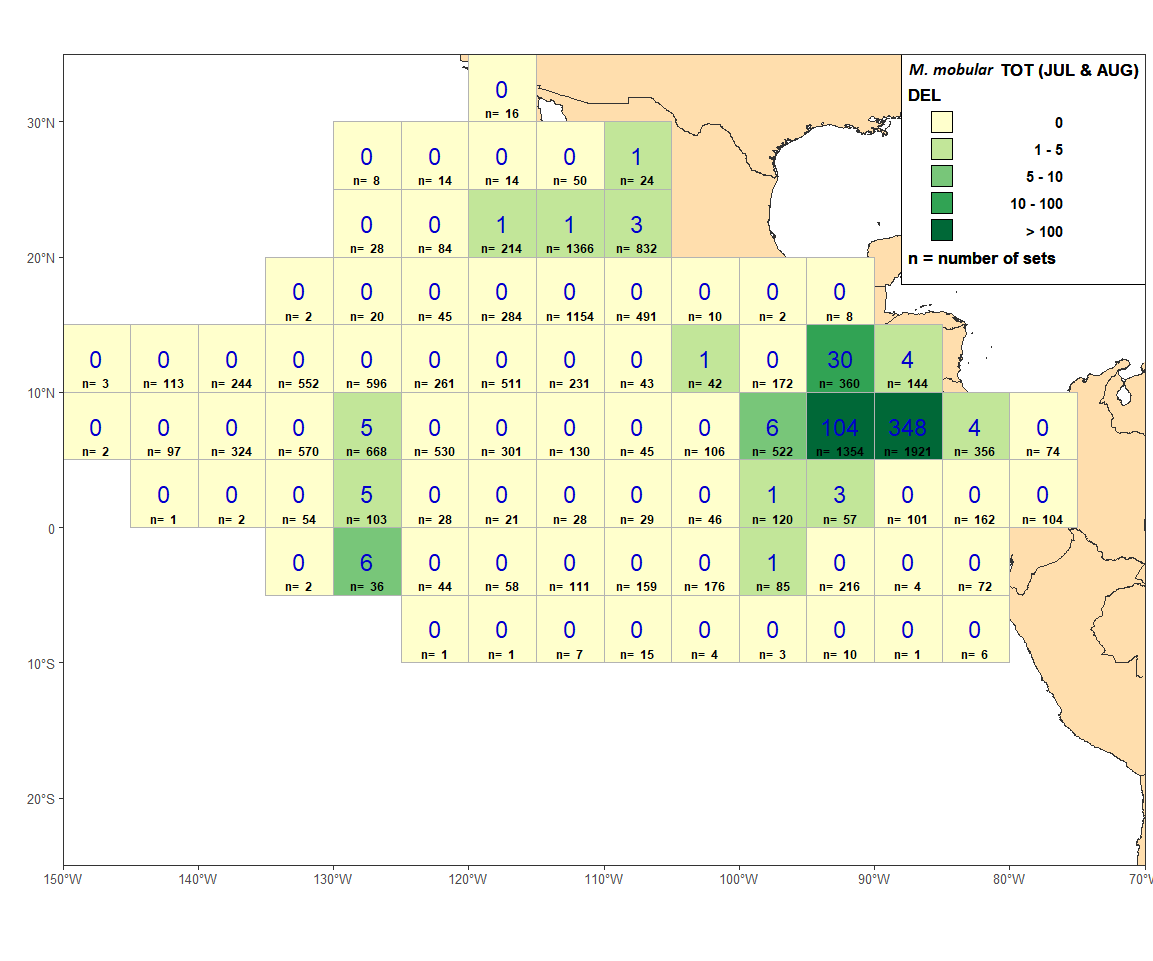

Supplement: S4 Fig — Dark green represents squares where highest number of individuals were observed (created by Marlon Román, IATTC). (DOCX) [file pone.0220854.s004.docx]
