# Supplementary material for: Environmental characteristics associated with the presence of the Spinetail devil ray (Mobula mobular) in the eastern tropical Pacific
Source: PLoS One. 2019 Aug 7;14(8):e0220854. doi: 10.1371/journal.pone.0220854 (PMC6685623; doi:10.1371/journal.pone.0220854)
Supplement: S1 Table — a,b. Example of script used to download environmental variables using Phyton and R routine code. (DOCX) [file pone.0220854.s005.docx]

**S1 Table a. Example of script used to download environmental variables using Phyton routine code.**

#************************Script to download CHL and O2 from 2003 to 2013************************

import datetime as dt

import subprocess

import time

import os

# ---------------------------------

# General Parameters

# ---------------------------------

# anything in bold must

# be modified by the user

#Python and Motu tools

python="C:\Python27\python.exe"

motu_cl="C:\motu-client-python\motu-client.py"

#Variables declaration

log_cmems="-u xxxx" #To adapt

pwd_cmems="xxxx " #To adapt

motu_sc="-m http://rancmems.mercator-ocean.fr/mis-gateway-servlet/Motu"

serv_id="-s http://purl.org/myocean/ontology/service/database#GLOBAL_REANALYSIS_BIO_001_018-TDS"

table_prod_id=["-d dataset-global-nahindcast-bio-001-018"]

# Date

yyyystart=2015

mmstart=01

ddstart=03

yyyyend=2016

mmend=01

ddend=02

hh= " 12:00:00"

st_date=dt.datetime(yyyystart,mmstart,ddstart) #Starting date

end_date=dt.datetime(yyyyend,mmend,ddend) #Ending date

# Area

xmin="-x -150"

xmax="-X 80"

ymin="-y -20"

ymax="-Y 30"

zmin="-z 0.494"

zmax="-Z 0.4942"

# Variables

table_var_cmd=["-v CHL -v nav_lon -v nav_lat"]

# Output files

out_path= "C:/Users//Desktop/" #To adapt

out_cmd="-o "+ out_path

table_data_type=["Chl"]

pre_name= "download_"

pre_file_cmd="-f "+ pre_name

# - - - - - - - - - - -

# Main Program

# - - - - - - - - - - -

# Counter

cpt=0

#Error Handle

try :

os.remove(out_path+"ficlog.txt")

except OSError:

print "Report Log File does not exist."

#While_Loop in order to launch again only unsuccessful requests

#while cpt<3:

for prod_id, var_cmd, datatype in zip(table_prod_id, table_var_cmd, table_data_type):

print "Processing request : "+datatype+st_date.strftime("%Y%m%d")+" - "+end_date.strftime("%Y%m%d")

date_cmd=" -t "+"\""+st_date.strftime("%Y-%m-%d"+hh)+"\""+" -T "+"\""+end_date.strftime("%Y-%m-%d"+hh)+"\""

end_file_cmd=datatype+st_date.strftime("%Y-%m-%d")+"_"+end_date.strftime("%Y-%m-%d")+".nc"

file_cmd=pre_file_cmd+end_file_cmd

ficout=pre_name+end_file_cmd

if not os.path.exists(out_path+ficout):

print os.path.exists(out_path+ficout)

cmd=' '.join([python,motu_cl,log_cmems,pwd_cmems,\

motu_sc,serv_id,prod_id, \

xmin,xmax,ymin,ymax,zmin,zmax, \

date_cmd, var_cmd,out_cmd,file_cmd])

print "## COMMAND ##"

print cmd

stspop=subprocess.Popen(cmd,shell=True).wait()

if stspop==0 :

print "Download : ",ficout, " OK"

time.sleep(60)

else :

if cpt==2:

f_log=open(out_path+"ficlog.txt","a")

f_log.write("Error : "+ficout+ " NOK\n")

f_log.close()

print "Report Log File created. Please check it out and launch again the script to download unsuccessful request(s)."

else:

print "Request : "+st_date.strftime("%Y%m%d")+ " - "+end_date.strftime("%Y%m%d")+" has already been downloaded and located in "+out_path+ficout

#cpt=cpt+1

#end=raw_input("Please press <Enter> to exit.")

#************************End of Script to download GLORYS month by month from 1993 to 2013************************

**S1 Table b. Example of script used to download environmental variables using R routine code.**

################# parameters for test

#################

#mydate="2005-02-03"

#dirnc="C:\\Users\\Desktop\\\\\\"

#longsample=2.883472

#latsample= -4.583472

#namenc="download_SST_2005-01-15_2013-12-15.nc"

getchlval_monthfile=function(mydate,latsample,longsample,dirnc,namenc)

{

library(ncdf4)

if(file.exists(paste(dirnc,namenc,sep=""))==FALSE){cellvalue=-888}else{

mync <- nc_open(paste(dirnc,namenc,sep=""))

print(mync)

year = as.numeric(format(as.Date(mydate), "%Y"))

year

mes=as.numeric(format(as.Date(mydate), "%m"))

mes

difmes = ((year-2005)*12) + mes

difmes

mesesinnc = length(ncvar_get(mync,"time"))

mesesinnc

if(difmes>mesesinnc){cellvalue=-777}else{

sst = ncvar_get( mync,"Temperature",start=c(1,1,1,difmes), count=c(923,209,1,1))

sst

rm(difmes)

x = ncvar_get( mync, "lon")

y = ncvar_get( mync, "lat")

difx=abs(x-longsample)

dify=abs(y-latsample)

idx <- which(difx == min(difx), arr.ind = TRUE)[1,1];idx

idy <- which(dify == min(dify), arr.ind = TRUE)[1,2];idy

cellvalue=sst[idx,idy]#####funciona

cellvalue

}############################################################ fin else 2

nc_close(mync)

rm(mync)

}############################################################ fin else 1

print(paste("cellvalue=",cellvalue))

return(cellvalue)

}

rm(list=ls(all=TRUE))

source("C:\\Users\\Nerea\\Desktop\\\\2015_getsstval_monthfile_corrected.R")

# read your table

t0<-read.csv("C:\\Users\\Nerea\\Desktop\\\\xx.csv",sep=",", header=T)

str(t0)

head(t0)

t0$latitude <- as.character(t0$latitude)

t0$latitude <- as.numeric(t0$latitude)

t0$date <- as.Date(t0$date)

t0$date

######################################################################################

###########

####

t2 <- t0

nrow(t2)

names(t2)

summary(t2)

rm(tout)

tout<-data.frame(

set_id=numeric(0),

date=as.Date(character()),

latitude=numeric(0),

longitude=numeric(0),

cellvalue=numeric(0),

stringsAsFactors = FALSE)

str(tout)

typeof(tout)

class(tout)

head(tout)

dirdata="C:\\Users\\Nerea\\Desktop\\\\"

dirdata

namesuf="download_SST_2005-01-15_2013-12-15"

namesuf

i=1

######

################################################################################### loop 1

for(i in 1:nrow(t2)){

sample<-t2[i,]

sample

rm(mydate)

mydate=format(strptime(sample$date,"%Y-%m-%d") )

namedate=format(strptime(sample$date,"%Y-%m-%d"),"%Y-%m")

mydate

rm(lat)

lat=sample$latitude

lat

rm(lon)

lon=sample$longitude

lon

myncname=paste(namesuf,".nc",sep="")

myncname

##################################################################### funcion

proces=getchlval_monthfile(mydate,lat,lon,dirdata,myncname)

#dev.off()###################################################################

rm(cellvalue)

cellvalue=proces[1]

tout[i,1]=sample$set_id

tout[i,2]=sample$date

tout[i,3]=sample$latitude

tout[i,4]=sample$longitude

tout[i,5]=cellvalue

rm(mydate)

}

nrow(tout)

# save

write.csv(tout,paste("C:/Users/Nerea/Desktop//sst_monthfile_2013_AO.csv",sep=""))
