# Supplementary material for: Environmental characteristics associated with the presence of the Spinetail devil ray (Mobula mobular) in the eastern tropical Pacific
Source: PLoS One. 2019 Aug 7;14(8):e0220854. doi: 10.1371/journal.pone.0220854 (PMC6685623; doi:10.1371/journal.pone.0220854)
Supplement: S2 Table — The piece-wise construction of the best model with each new variable improving the AIC value (below). (DOCX) [file pone.0220854.s006.docx]

**S2 Table. Final candidate Generalized Additive Models (GAMs) with the corresponded Akaike Information Criteria (AIC) values and the variables selected for each model (up). The piece-wise construction of the best model with each new variable improving the AIC value (below).**

| **GAM** | **AIC** | **Variables** |
| --- | --- | --- |
| **Option 1** | **7603.036** | **Latitude*Longitude + Type + Month + Chl + SSH+ O2 +Ni** |
| Option 2 | 7606.235 | Latitude + Distance + Type + Month +Chl + Ni +O2 +SSH +heading |
| Option 3 | 7658.977 | Distance + Type + Month + Chl + Ni + O2 + SSH + Vel + Heading |
| Option 4 | 7659.616 | Distance + Type + Month + Chl + Ni + O2 + SSH + Ke + Heading |
| Option 5 | 8485.276 | Longitude + Year + Type + Chl + Ni+ SST + Vel + Heading |
| Option 6 | 8486.127 | Longitude + Year + Type + Chl + Ni + SST + Ke + Heading |
| Option 7 | 8489.774 | Latitude + Distance + Year + Type + Chl + Ni + SST + Vel + Heading |
| Option 8 | 8479.344 | Latitude + Distance + Year + Type + Chl + Ni + SST + Ke + Heading |
| Option 9 | 8556.972 | Distance + Depth + Year + Type + Chl + Ni + SST + Vel + Heading |
| Option 10 | 8561.238 | Distance + Depth + Year + Type + Chl + Ni + SST + Ke + Heading |
| Option 11 | 8765.435 | Latitude + Longitude + Year + Type + Chl + Ni + SST |
| Option 12 | 8970.222 | Latitude + Longitude + Type + Month + Chl + Ni+ O2 |
| Option 13 | 9031.129 | Longitude + Type + Month + Chl + Ni + O2 |

| **Variables** | **AIC** |
| --- | --- |
| Latitude*Longitude | 10479.08 |
| Latitude*Longitude + Type | 8953.787 |
| Latitude*Longitude + Type + Month | 8946.356 |
| Latitude*Longitude + Type + Month + Chl | 8891.562 |
| Latitude*Longitude + Type + Month + Chl + SSH | 7658.689 |
| Latitude*Longitude + Type + Month + Chl + SSH + O2 | 7638.566 |
| Latitude*Longitude + Type + Month + Chl + SSH + O2 + Ni | 7603.036 |
